# Integrating Machine Learning with Hybrid and Surrogate Models to Accelerate Multiscale Modeling of Acute Respiratory Infections

**DOI:** 10.3390/v17121541

**Published:** 2025-11-25

**Authors:** Andrey Korzin, Maria Koshkareva, Vasiliy Leonenko

**Affiliations:** 1 Research Center “Strong Artificial Intelligence in Industry”, ITMO University, Saint Petersburg 199034, Russia; corzin.an@gmail.com (A.K.); koshk.mp@gmail.com (M.K.); 2 Laboratory of Influenza and Acute Respiratory Illness Epidemiology, Smorodintsev Research Institute of Influenza, Saint Petersburg 197022, Russia

**Keywords:** machine learning, hybrid modeling, surrogate modeling, mathematical epidemiology, influenza, COVID-19

## Abstract

Accurate, efficient, and explainable modeling of the dynamics of acute respiratory infections (ARIs) remains, in many aspects, a significant challenge. While compartmental models such as SIR (Susceptible–Infected–Recovered) remain widely used for that purpose due to their simplicity, they cannot capture the complicated multiscale nature of disease progression which unites individual-level interactions affecting the initial phase of an outbreak and mass action laws governing the disease transmission in its general phase. Individual-based models (IBMs) offer a detailed representation capable of capturing these transmission nuances but have high computational demands. In this work, we explore hybrid and surrogate approaches to accelerate forecasting of acute respiratory infection dynamics performed via detailed epidemic models. The hybrid approach combines IBMs and compartmental models, dynamically switching between them with the help of statistical and ML-based methods. The surrogate approach, on the other hand, replaces IBM simulations with trained autoencoder approximations. Our results demonstrate that the usage of machine learning techniques and hybrid modeling allows us to obtain a significant speed–up compared to the original individual-based model—up to 1.6–2 times for the hybrid approach and up to $10^{4}$ times in case of a surrogate model—without compromising accuracy. Although the suggested approaches cannot fully replace the original model, under certain scenarios they make forecasting with fine-grained epidemic models much more feasible for real-time use in epidemic surveillance.

## 1. Introduction

Accurate, efficient, and explainable city-scale forecasting of epidemic dynamics remains, in many aspects, a significant challenge. This is especially the case for urban areas, which are increasingly becoming the epicenters of both technological innovation and epidemiological vulnerability. Advances in surveillance, modeling, and policy reorients epidemic response from passive reaction to proactive adaptation based on real-time disease forecasting. Such a concept, however compelling, requires appropriate data management and adequate highly detailed disease propagation models. Given the wide range of available approaches [1], selecting an appropriate method that is both fast and accurate remains a challenging task.

In practice, public health agencies predominantly rely on forecasts made with compartmental models such as the SIR (Susceptible–Infected–Recovered) model, despite their well-known limitations in capturing the complexity of disease transmission. SIR-type models [2,3], though conceptually simple and a century old, served as the backbone of the COVID-19 pandemic response [4,5]. To a casual observer, the continued reliance on such a simplistic framework might appear outdated or even counterintuitive. The enduring popularity of SIR models can be attributed to their simplicity, ease of implementation, and a minimal set of parameters, which facilitates rapid calibration and leads to reduced uncertainty in model outputs, albeit often at the cost of potential bias.

Among the reasons against using SIR models as the only forecasting tool in epidemic surveillance is their inability to explicitly account for connectivity of individuals and stochasticity of their behavior. Connectivity, especially in early outbreak stages, is more closely correlated with disease spread than density per se. Accounting for stochasticity is especially important in the early phases of an outbreak, when individual-level randomness plays a large role and data remain sparse. To meet the mentioned needs, lightweight stochastic models were proven effective [6,7]. For example, during the modeling of COVID-19 transmission aboard the Diamond Princess cruise ship [8], researchers used a variety of probabilistic approaches, including branching processes [9], Markov chain models [10], and chain binomial models [11]. These models were chosen for their ability to flexibly handle various transmission assumptions, track infection chains at the individual level, and operate with high computational efficiency.

More sophisticated forecasting techniques, such as those relying on agent-based models (ABMs) [12,13,14] and network-based models [15,16], are capable of capturing heterogeneity in populations and complex interaction patterns. These methods offer richer insights into transmission dynamics, as they are capable of taking into account individual behaviors, mobility patterns, and contact networks. However, those advantages come at a price. The highly detailed models pose significant demands in terms of data requirements and computational resources. As a result, deploying individual-based models (IBMs) for real-time epidemic forecasting or for timely public health decision-making is often impractical. This limitation has even led some researchers to question whether ABMs are suitable for predictive use at all [17].

In this work, we argue that multiscale individual-based models could play a more active role in epidemic forecasting, provided they are capable of rapid calibration to real-time data and operate under constrained computational resources. The aim of our study is to address the limitations that currently hinder the use of IBMs in fast-paced decision-making environments. Specifically, we focus on improving computational efficiency of forecasting frameworks through the use of hybrid and surrogate approaches and evaluate their performance using a network-based epidemic model of acute respiratory disease dynamics as a baseline.

## 2. Related Works

### 2.1. Hybrid Modeling

Hybrid models of epidemic outbreaks come in great variety, and the term “hybrid” is interpreted differently depending on the specific implementation. In recent years, hybrid approaches that combine mechanistic models and machine learning methods are especially popular [18]. Physics-/epidemiology-informed neural networks (PINNs/EAAMs) embed compartmental dynamics in neural architectures to fit time-varying parameters and forecast short horizons with calibrated uncertainty and mechanistic interpretability [19]. Variants include SIR-INN, which trains a single PINN once on synthetic epidemics and then performs fast weekly Bayesian updates for influenza forecasts (4–10 weeks ahead) without retraining [20], and “informed” architectures that learn mappings from interventions and exogenous signals into epidemiological dynamics within the SEIR models [21]. Spatial hybrids combine meta-population SEIR with deep learning to estimate mobility-dependent parameters and improve $I_{t}$ forecasts relative to pure ML or pure ODE baselines [22]. ODE–ABM model-predictive control (MPC) loops compute continuous transmission targets with an epidemiological ODE and then map them to discrete, implementable non-pharmaceutical intervention (NPIs)—masking, closures, testing—via a learned lookup table, updating every 21–30 days and adaptively re-scaling when variants change transmissibility [23]. Compared to traditional methods, ML methods demonstrated significant advantages in COVID-19 prediction, especially hybrid modeling strategies, which showed great potential in optimizing accuracy [24]. Comprehensive reviews on coupling explanatory models with ML methods can be found in [25,26].

In this study, we regard a subset of hybrid approaches that aim to combine the fine-grained detail of IBMs with the computational efficiency of compartmental models. According to [27], hybrid approaches generally fall into two categories: The first involves modeling different components of the system using different approaches—some parts are represented with IBMs, while others use compartmental models. The second category employs a dynamic switching mechanism that alternates between IBM and compartmental models based on specific condition, such as reaching a case threshold or relying on other modeling parameters that indicate the moment when the law of big numbers starts working and a population can be assumed homogeneous.

Among the earlier works, ref. [28] introduced a method that switches between an ABM and an SEIR model. In their framework, switching is triggered when the number of infected individuals reaches a predefined absolute count. However, applying a fixed number as a switching threshold becomes problematic in urban areas with varying population sizes. To address this, the authors of [28] proposed using the stabilization of the transmission parameter β as a dynamic proxy for population-level homogeneity, offering a more adaptive switching condition.

In [29], the authors presented a hybrid approach to simulate outbreak dynamics at two spatial scales: town and county. They concluded that initiating the switch at the town level offers the best trade-off between computational efficiency and accuracy in reproducing results from an ABM. This approach was later adapted to the context of COVID-19 outbreaks in Irish counties [30].

In [31], the authors developed an approach to predict the daily transmission rate using statistical and ML methods. The predicted transmission rate was then used within SIRV to forecast the number of confirmed COVID-19 cases for macro-level data (Africa, Germany, and Netherlands). The study concluded that nesting SIRV with these methods produces more accurate forecasts than the methods alone. This study is especially interesting, as it is based on predicting the form of transmission rate function, which is close to the idea we employ in this paper.

The authors of the current paper have previously investigated hybrid approaches for modeling multistrain influenza epidemics in a synthetic population of Saint Petersburg, Russia [32]. In a related research, we also explored different strategies for estimating disease transmission rates to facilitate accurate switching between submodels within a hybrid approach [33].

### 2.2. Surrogate Modeling

Surrogate models—also known as emulators or meta-models—are simplified models trained to approximate the behavior of computationally expensive simulations. In recent years, neural networks have gained traction as surrogate models due to their ability to capture complex, highly nonlinear relationships. Surrogates are particularly useful for accelerating inference, enabling real-time forecasting, and performing uncertainty quantification.

In epidemiology, surrogate modeling has been used for the calibration of compartmental models such as SIR and SEIR. For example, ref. [34] introduced a deep neural network-based surrogate modeling (DNN-SM) approach to optimize parameter estimation. Their method was applied to calibrate three structurally distinct SIR-type models (SIR, SEIR, and SEPADR) to both short- and long-term COVID-19 datasets from several European countries. Their surrogate models achieved high forecasting accuracy and delivered a roughly tenfold acceleration compared to traditional ODE solvers.

Surrogate approaches have also been deployed to emulate more complex epidemic models, including those based on probabilistic cellular automata (PCA). In [35], a deep learning-based surrogate significantly reduced computational cost while maintaining a high fidelity to the original PCA-based epidemic model.

Surrogates become even more advantageous when applied to detailed models such as network models and ABMs. In [36], nine surrogate methods were compared in replicating the behavior of the “Linked Lives” ABM, a model developed to evaluate social care policies in the UK. The study found that artificial neural networks (ANNs) and gradient-boosted trees generally outperformed Gaussian process (GP) surrogates, which are traditionally popular in surrogate modeling [37,38,39]. In [40], a graph neural network (GNN) serves as a surrogate for a spatially and demographically resolved metapopulation simulation, accelerating its evaluation by up to 28,670 times compared to the original SIRD mechanistic model. In [41], the researchers applied surrogate modeling to two ABMs of acute respiratory infections and compared their performance with several calibration algorithms, including Markov chain Monte Carlo (MCMC), particle swarm optimization (PSO), genetic algorithms (GAs), and chaos game optimization. The study emphasized that while surrogate models can significantly accelerate simulations and eliminate the opaqueness of “black-box” ML models by retaining interpretable parameters, they require substantial computational resources for training due to the necessity of extensive parameter space exploration.

Surrogate models can also be used to complement rather than fully replace detailed models, particularly to enhance calibration processes. In [42], a novel surrogate-based calibration approach was proposed for the Epicast ABM, targeting multiple metropolitan areas in the United States. Their neural network-based surrogate was capable of accurately reproducing the behavior of the ABM across locations that were not included in the training set, showcasing generalization capability and computational efficiency. In [43], the authors present an adaptive framework that couples agent-based models (ABMs) with surrogate-assisted, derivative-free optimization to efficiently calibrate infectious-disease ABMs. The framework’s performance is demonstrated using synthetic and real COVID-19 data (for South Africa). The study evaluated several sampling methods alongside multiple surrogate types, including XGBoost, Decision Tree, and Support Vector Machines.

A comprehensive review of surrogate modeling for ABMs is offered in [44]. The review discusses surrogate-assisted methodologies across biological and biomedical applications, covering statistical, mechanistic, and ML-based approaches. The study emphasizes the high computational cost of parameter estimation and uncertainty quantification in ABMs. These issues often hinder their use in real-time epidemic surveillance and decision-support systems. Ref. [44] supports the growing trend toward surrogate models that balance accuracy with computational efficiency.

Beyond direct surrogation, several auxiliary techniques have been developed to assist ABM calibration. One such method is ML-ABC (Machine Learning-enhanced Approximate Bayesian Computation), proposed in [45]. Applied to the calibration of the open-source COVASIM COVID-19 model [46], this approach reduced parameter optimization time by approximately 52% during the first epidemic wave and 33% during the second wave.

In our current work, we explore two methodologies for reducing the computational burden of detailed simulations by replacing the most intensive calculations with approximations of disease trajectories. In the hybrid approach, a detailed model is supplemented by a simple compartmental model, with switching governed by an ML model that tracks transmission rate dynamics. In the surrogate approach, the entire detailed model is replaced by a “black-box” approximation trained on the outputs of the original simulation.

The proposed surrogate models build upon our previous research [47], where we used autoencoders to approximate an ABM of influenza spread in a synthetic population of Saint Petersburg, Russia. While the method itself proved effective, the choice of the original ABM raised concerns regarding data quality. Specifically, the lack of well-validated mobility data in Russian cities introduces significant uncertainty when employing complex behavioral patterns. To address this issue, we adopted a simplified network model that retains the same synthetic population but omits detailed contact layers such as workplaces, households, and schools. Instead, transmission events are simulated via graph connections between nodes.

Using well-known network topologies improves the generality of our speed-up results and enables the application of the proposed techniques beyond infectious disease modeling, including use cases such as computer virus propagation and the spread of information or rumors.

## 3. Materials and Methods

### 3.1. Epidemic Models

We employed the SEIR models in both the compartmental and network model formulations. The parameters and variables are summarized in Table 1. Infection transmission rates are denoted as $\beta_{c}$ for the compartmental model and $\beta_{n}$ for the network model.

The assumptions for the epidemic models are as follows:

- The modeled population represents one large metropolitan area without strong clusterization (i.e., the city districts are well connected with each other).
- The contact patterns of individuals in a given city does not exhibit substantial changes in the course of several years, so the topology of contact networks might be approximately assessed before the outbreak.
- The population size is constant: we do not consider mortality, migration, births.
- The contact network is fixed and not influenced by an epidemic (i.e., network edges are not added or removed during the simulation).
- The individuals do not differ (i.e., have the same transmission rate and latent and infectious periods).
- The modeled infection is caused by a single virus which does not change its characteristics during the simulation.

#### 3.1.1. Network Model

The original SEIR network model, which serves as a baseline in this study, is based on our earlier works related to simulating dynamics of influenza and COVID-19 in Russian cities [48,49].

**Figure 1:**
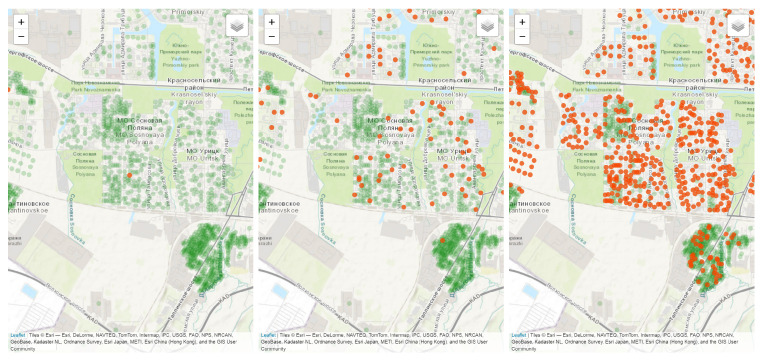
Example of epidemic dynamics in a network simulation in time moments t=0,5,10. Red dots represent infected households and green dots represent susceptible or exposed households.

The model can be initialized either with a fixed number of nodes and a predefined topology, or with a synthetic population that incorporates demographic and social characteristics. The methods of constructing synthetic populations and transforming them into network representation are described in other papers. This technique allows simulating epidemic dynamics considering specific urban social interactions. The example of usage of synthetic population as a base for disease propagation simulation on a network is shown in Figure 1, which demonstrates the outbreak in a synthetic population of Saint Petersburg, second largest Russian city, with over 5 million inhabitants. The red dots represent infected households and the green dots represent susceptible or exposed households on day *t*.

In this representation, individuals correspond to nodes, and edges represent potential contacts through which transmission may occur. The population structure is, therefore, heterogeneous, since not all individuals are connected with each other.

The stochastic process is simulated using the Gillespie algorithm. At each step we generate transition times for next events from exponential distributions with parameters $\beta_{n}$, γ, and δ. The event with the earliest time is executed, triggering the corresponding compartment transition and updating the system state. The detailed description of the algorithm is provided in [50], and its application to our case is described in Appendix C; the implementation is available in the Epidemics on Networks library [51].

Contact network graphs constructed from synthetic populations provide the most reliable representations of patterns of daily human interactions, but such datasets are not always readily accessible and require validation. A fast alternative solution is the usage of standard topologies, such as Barabási–Albert or Watts–Strogatz topologies, as an approximation for the real interaction structure [52,53].

#### 3.1.2. Compartmental Model

The SEIR model is employed as a submodel in the hybrid approach, capturing both latent and infectious periods of transmission. It is described by the following system of difference equations:

(1) $$\begin{array}{cc} \; & S_{t+1}=S_{t}-\beta_{c}S_{t}I_{t},\\ \; & E_{t+1}=E_{t}+\beta_{c}S_{t}I_{t}-\gamma E_{t},\\ \; & I_{t+1}=I_{t}+\gamma E_{t}-\delta I_{t},\\ \; & R_{t+1}=R_{t}+\delta I_{t},\\ \; & S_{0}\geq 0,\;E_{0}\geq 0,\;I_{0}\geq 0,\;R_{0}\geq 0,\;R_{0}=\alpha \times N,\\ \; & S_{0}+E_{0}+I_{0}+R_{0}=N. \end{array}$$

The parameters are specified in Table 1.

### 3.2. Data Generation

To compare disease transmission dynamics across different contact patterns, we generated two graphs representing two differently connected populations—a Barabási–Albert network where each new node connects to five existing nodes (m=5) and a small-world network topology with the following parameters: the number of neighbours in ring topology k=5 and the probability of rewiring of each edge p=0.1. For implementation details, see NetworkX documentation for barabasi_albert_graph and watts_strogatz_graph [54]. These topologies capture heterogeneous contact patterns and hub formation consistent with real-world social structures. Once generated, the networks were fixed and did not change from simulation to simulation.

To train machine learning models used for hybrid and surrogate approaches, we generated synthetic incidence datasets representing an epidemic ARI outbreak by simulating the network model on the two networks described above. All the parameter values are summarized in Table 2. The parameters that reflect biological properties of the disease (e.g., durations of the latent and infectious periods) were fixed. For the network model, α∈[0.2,1] and $\beta_{n}\in \left[0.1,1\right]$ were varied in steps of 0.01. For each $\left(\alpha ,\beta_{n}\right)$ pair, 10 simulations were run over T=100 days for the Barabási–Albert topology and over T=350 days for the small-world topology; the network size was set to $N=10^{5}$. Each dataset consisted of 72,000 epidemic trajectories.

The model output used for calibration to epidemic data is the daily incidence $I^{\left(new\right)}\left(t\right)$—the number of new symptomatic cases on day *t*. This is the number of transitions E → I on day *t*, i.e., the analog of transition term $\gamma E_{t}$ in the SEIR model. Thus, the daily incidence can be approximated by the following formula:

(2) $$I^{\left(new\right)}\left(t\right)=\left(E_{t}-E_{t+1}\right)-\left(S_{t+1}-S_{t}\right).$$

**Figure 2:**
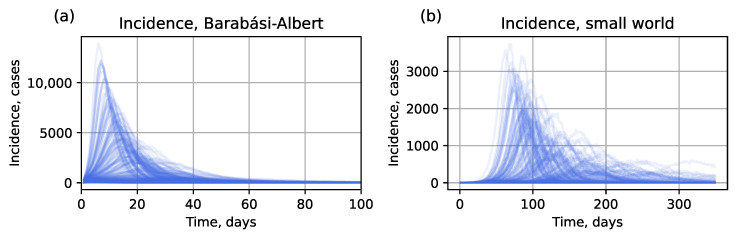
A 10% sample from the test dataset generated with parameters α∈[0.2;1] and $\beta_{n}\in \left[0.1;1\right]$: (**a**) incidence curves for Barabási–Albert topology, (**b**) incidence curves for small-world topology.

**Figure 3:**
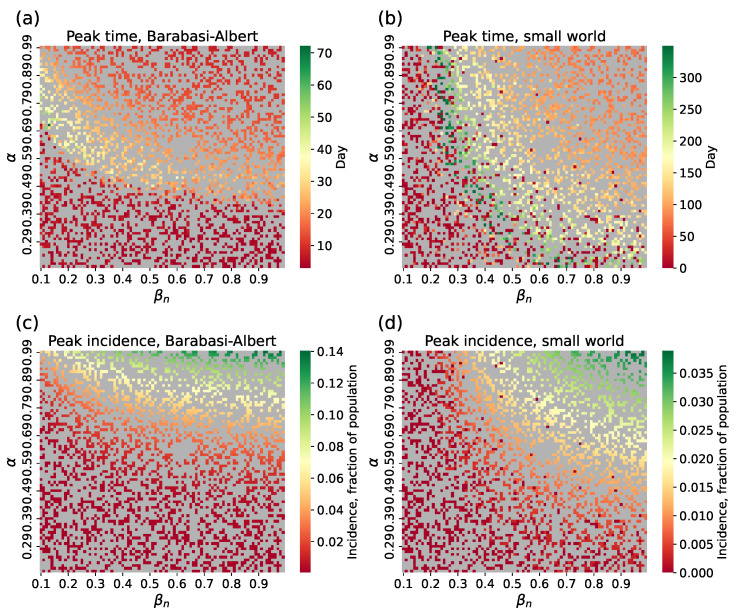
Distribution of (**a**,**b**) peak time and (**c**,**d**) peak incidence for synthetic incidence curves for Barabási–Albert and small-world topologies; gray cells represent train samples.

We had two datasets in total: a full dataset (10 simulations per $\left(\alpha ,\beta_{n}\right)$ pair) for the surrogate approach with interval estimation, and a reduced dataset (1 simulation per pair) for the hybrid and point-estimation surrogate approaches. Each dataset was split into training, validation, and test sets in a 3:1:2 ratio. Examples of the simulated curves are provided in Figure 2; peak time and peak incidence distributions are shown in Figure 3. It can be seen that the small-world topology corresponds to a different typical shape of incidence curves compared to the Barabási–Albert network. Particularly, for the same parameter value intervals, it shows a low and delayed epidemic peak. This can be explained by lower speed of infection propagation through the network. Consequentially, in two cities of the same size but with different connectivity patterns, one can witness completely different outbreaks caused by the same virus strain (similar results were shown for epidemic propagation in synthetic populations in [48]), which should be considered in predictive models to avoid loss of accuracy. Considering the presence of a long-term immunity formed as a result of consecutive outbreaks [55] makes the epidemic dynamics even more complicated in terms of modeling and prediction.

### 3.3. Hybrid Approach

Our hybrid approach functions according to the following algorithm:

1. Initialize an IBM with parameters $\left(\alpha ,\beta_{n}\right)$.
2. Identify the switch time $t_{switch}$ when the infected population can be assumed homogeneous, so the perfect mixing assumption is considered to be satisfied, making the SEIR model applicable. The switch time is determined by sufficient infection prevalence to meet the well-mixed assumption; $I_{t}/N>\epsilon$.
3. Switch to the SEIR model initialized from compartments $\left(S_{t},E_{t},I_{t},R_{t}\right)$ and parameters $\left(\beta_{c},\gamma ,\delta \right)$.

A key component determining the accuracy of the hybrid approach (how well the compartmental model approximates the IBM) is the correct calculation of $\beta_{c}$ [33]. While the parameter values γ and δ are interchangeable between the submodels, estimating $\beta_{c}$ is challenging, because it cannot be directly taken from the network model parameters. In the network submodel, the infection dynamics is defined by the contact network topology and the constant value of transmission intensity. In contrast, the infection transmission in the compartmental submodel is solely governed by the transmission rate. Consequently, the parameters $\beta_{n}$ and $\beta_{c}$ have different meanings.

**Figure 4:**
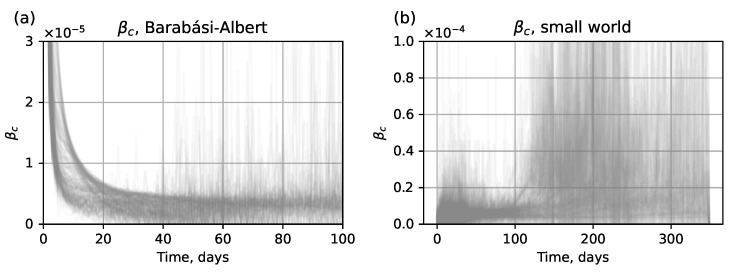
A 10% sample from the test dataset generated with parameters α∈[0.2;1] and $\beta_{n}\in \left[0.1;1\right]$: (**a**) ${\hat{\beta }}_{c}\left(t\right)$ curves for Barabási–Albert topology, (**b**) ${\hat{\beta }}_{c}\left(t\right)$ curves for small-world topology.

There are several methods to obtain the value of $\beta_{c}$ for SEIR submodel:

- Analytical method. Assuming $\beta_{c}=f\left(\beta_{n}\right)=const$, find f(…) based on the network topology. This method works only for $\beta_{c}=const$, which limits the accuracy of the hybrid model. In such cases the SEIR submodel is generally unable to closely approximate the IBM [33].
- Numerical method. Assess the value of $\beta_{c}$ using the approximate values of ${\hat{\beta }}_{c}={\hat{\beta }}_{c}\left(t\right)$ derived from the output of the network submodel. The estimated compartmental transmission rate ${\hat{\beta }}_{c}\left(t\right)$ can be found via a formula derived from Equation (1) of the ODE system (1), which gives
  (3) $${\hat{\beta }}_{c}\left(t\right)=-\frac{S_{t+1}-S_{t}}{S_{t}\times I_{t}},t\leq t_{switch}.$$
  The typical form of ${\hat{\beta }}_{c}\left(t\right)$ curves obtained from the test dataset is shown in Figure 4. Here we show ${\hat{\beta }}_{c}\left(t\right)$ for $t\in \bar{1,T}$; however, in an actual simulation run, this data is available only for $t\leq t_{switch}$, before the network submodel is replaced by the SEIR submodel.

We evaluated multiple $\beta_{c}$ estimation strategies:

- Statistical: Last ${\hat{\beta }}_{c}\left(t\right)$ value observed on the switch day, i.e., ${\hat{\beta }}_{c}\left(t_{switch}\right)$; cumulative average for ${\hat{\beta }}_{c}\left(t\right),t\leq t_{switch}$, or median of ${\hat{\beta }}_{c}\left(t\right),t\leq T$ trajectories from the train set.
- ML: Polynomial regression (third order with L2 regularization), and a Long Short-Term Memory network (LSTM) trained on ${\hat{\beta }}_{c}\left(t\right)$.

We trained four ML models for the hybrid approach: LSTM for Barabasi–Albert, LSTM for small world, regression for Barabasi–Albert, and regression for small world. Each model was trained on the training set (3600 trajectories) and checked on the validation set (1200 trajectories); the final metrics were calculated on the test set (2400 trajectories). For training, we used $\beta_{c}\left(t\right)$ from each of the 3600 train trajectories. Each ML method consequentially predicted the next $\beta_{c}\left(t\right)$ value based on a window of *w* most recent values. Once all future $\beta_{c}\left(t\right)$ values were predicted, the SEIR model was initialized with these predicted $\beta_{c}\left(t\right)$.

The LSTM consisted of two layers with 64 neurons each, with dropout rates 0.2 and 0.3, respectively. The optimizer was RMSprop with initial learning rate 0.001 and a schedule to multiply the learning rate by 0.1 after 30 epochs. The training was performed with a batch size of 64 for 100 epochs with early stopping, if validation loss did not improve after 15 epochs.

### 3.4. Surrogate Approach

The surrogate approach entails developing a simplified and computationally efficient model that replicates the dynamics of a more complex and resource-intensive simulation. The proposed surrogate models build upon our previous research [47].

**Figure 5:**
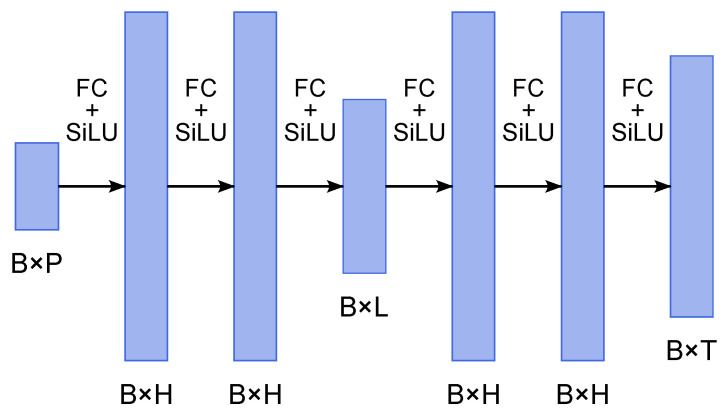
Architecture of the autoencoder model. Encoder and decoder consist of three fully connected layers with SiLU activation. Input size is B×P, where B=16 is the batch size and P=2 for parameters $\left(\alpha ,\beta_{n}\right)$. Hidden dimension H=256 and latent dimension L=32 were obtained via grid search.

The autoencoder (Figure 5) consists of the following:

- Encoder: Three fully connected layers with SiLU activations, mapping inputs of size B×P to latent space B×L, where *B* is the batch size, *P* is the number of input parameters, *L* is the latent dimension;
- Decoder: Three fully connected layers with SiLU activations, mapping the latent space back to time series of size B×T.

Hyperparameters were optimized by grid search, resulting in hidden dimension H=256 and latent size L=32. Training employed mean squared error (MSE) loss. Although incidence trajectories were selected as the main model output, this approach can be adapted to reconstruct trajectories of $S_{t}$, $E_{t}$, and $R_{t}$, which allows to estimate additional epidemic indicators, such as the effective reproduction number.

We implemented two surrogate modeling strategies. The surrogate approach with point estimation uses a set of trajectories—one for each pair of parameters $\left(\alpha ,\beta_{n}\right)$—and outputs a corresponding incidence trajectory, so it is fully interchangeable with the original network model in terms of input and output. Its limitation is the lack of stochasticity, as it produces only one deterministic output for each input.

To overcome this limitation, we developed the surrogate approach with interval estimation. For each $\left(\alpha ,\beta_{n}\right)$ pair, the model outputs three time series: lower estimate ${\hat{l}}_{t}$, mean ${\hat{\mu }}_{t}$, and higher estimate ${\hat{u}}_{t}$. The targets are $l_{t}$, $\mu_{t}$, and $u_{t}$, calculated as the minimum, mean, and maximum of ten network model trajectories from training set. We formed $y_{t}$ as a concatenation of $l_{t}$, $\mu_{t}$, and $u_{t}$, i.e., an array of size 3T. A custom loss function was used to enforce the inequality ${\hat{l}}_{t}\leq {\hat{\mu }}_{t}\leq {\hat{u}}_{t}$. Let ${\hat{y}}_{t}$ be the concatenation of ${\hat{l}}_{t},{\hat{\mu }}_{t}$, and ${\hat{u}}_{t}$. The equation for the loss function has the following form:

(4) $$\begin{array}{cc} L=\;\underset{\mathrm{MSE}\;\mathrm{for}\;\mathrm{concatenated}\;\mathrm{array}}{\underset{︸}{\frac{1}{3T}\sum_{t=1}^{3T}{\left({\hat{y}}_{t}-y_{t}\right)}^{2}}}+ & \underset{\mathrm{Penalty}\;\mathrm{if}\;\mathrm{mean}\;\mathrm{outside}\;\mathrm{bounds}}{\underset{︸}{\frac{1}{T}\sum_{t=1}^{T}\left[\max\left(0,{\hat{\ell }}_{t}-{\hat{\mu }}_{t}\right)+\max\left(0,{\hat{\mu }}_{t}-{\hat{u}}_{t}\right)\right]}}\\ \; & +\underset{\mathrm{Penalty}\;\mathrm{if}\;\mathrm{upper}\;\mathrm{bound}\;\mathrm{not}\;\mathrm{higher}\;\mathrm{than}\;\mathrm{lower}\;\mathrm{bound}}{\underset{︸}{\frac{1}{T}\sum_{t=1}^{T}\max\left(0,{\hat{\ell }}_{t}-{\hat{u}}_{t}\right).}} \end{array}$$

Here the second term is zero if the predicted mean ${\hat{\mu }}_{t}$ lies within the bounds $\left[{\hat{l}}_{t},{\hat{u}}_{t}\right]$, and positive otherwise. The third term is zero if the predicted upper bound ${\hat{u}}_{t}$ is greater than the predicted lower bound ${\hat{l}}_{t}$, and positive otherwise. As a result, any output that violates these constraints is penalized.

## 4. Results

### 4.1. Numerical Experiments with Simulated Data

#### 4.1.1. Hybrid Approach

The aim of this experiment was to evaluate the ability of the SEIR model to approximate network model dynamics with different methods of $\beta_{c}$ estimation. For each test trajectory, we determined the switching time, estimated future values of $\beta_{c}$, and continued the simulation using the SEIR.

The minimum allowed switch day was w=4, corresponding to the window size used in estimation methods. If the switch condition was not met, the switch day defaulted to w=4. The switch condition was defined as reaching I/N=0.05, approximating the midpoint of the epidemic’s rising phase (left half-wave). The resulting accuracy of incidence assessment for the Barabási–Albert and small-world network models is shown in Table 3 and Table 4, respectively. As shown in Table 3, using other percentages produces similar RMSE values. Switching later reduces error, since the trajectory is closer to its peak, but also increases computational cost of the network simulation. The cumulative average method showed the highest errors, because taking the average of declining $\beta_{c}$ values results in an overestimated value, and was excluded from further analysis. As shown in Table 4, the hybrid approach is equally effective for simulations on networks with small-world topology, albeit the ranking of best methods is different. An outbreak develops slower (Figure 2), therefore the switch occurs later, which gives $\beta_{c}$ time to stabilize. In these conditions, cumulative average method seems to provide a good approximation for $\beta_{c}$.

**Figure 6:**
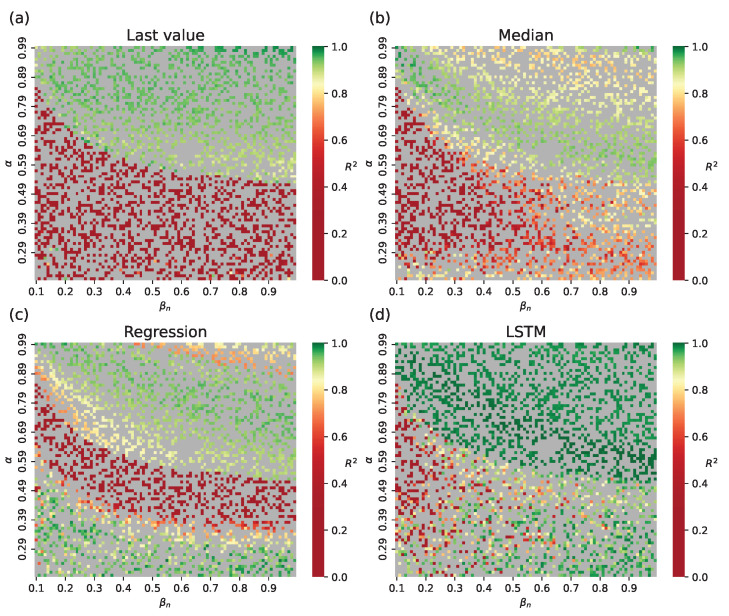
$R^{2}$ values for incidence trajectories under different $\beta_{c}$ estimation methods for the hybrid approach, Barabási–Albert topology: (**a**) last observed value of ${\hat{\beta }}_{c}$, (**b**) median of ${\hat{\beta }}_{c}$ from the train set, (**c**) polynomial regression trained on ${\hat{\beta }}_{c}$, (**d**) LSTM trained on ${\hat{\beta }}_{c}$. Gray cells indicate training samples.

**Figure 7:**
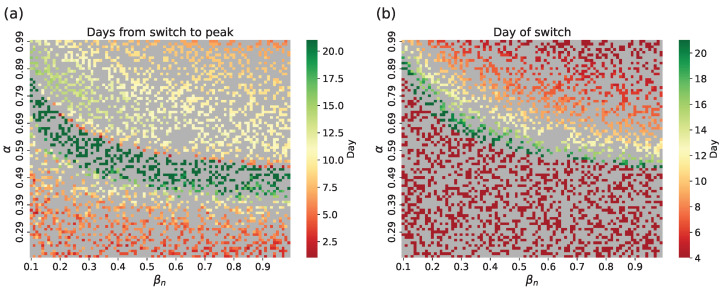
Switching behavior for test samples: (**a**) difference between epidemic peak time and day of switch; (**b**) distribution of switch days across all runs. Gray cells indicate training samples.

**Figure A1:**
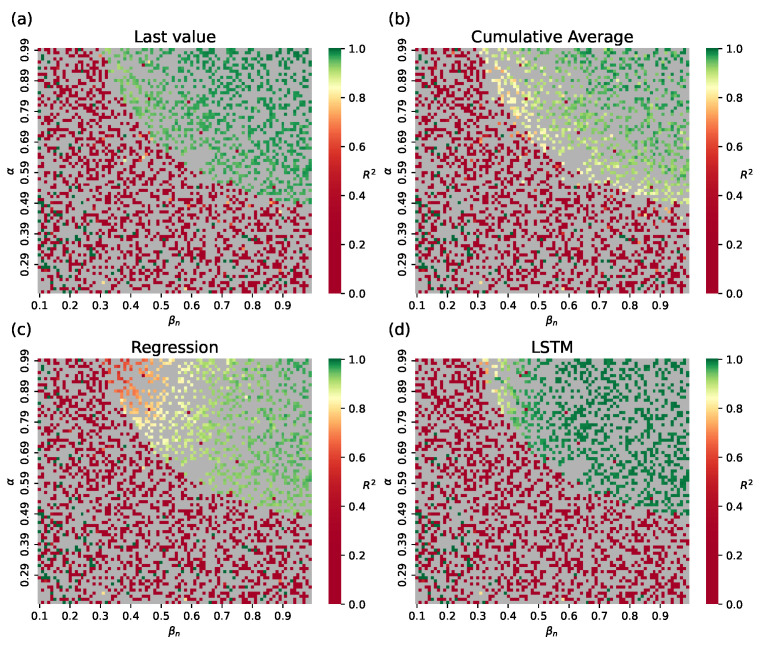
$R^{2}$ values for incidence trajectories under different $\beta_{c}$ estimation methods for the hybrid approach (switch condition I/N=0.05), small-world topology: (**a**) last observed value of ${\hat{\beta }}_{c}$, (**b**) cumulative average of ${\hat{\beta }}_{c}$, (**c**) polynomial regression trained on ${\hat{\beta }}_{c}$, (**d**) LSTM trained on ${\hat{\beta }}_{c}$. Gray cells indicate training samples.

Figure 6 shows the $R^{2}$ scores for full incidence trajectories across test samples for switch condition I/N=0.05 (the results for the small-world topology are shown in Figure A1). Since the hybrid approach approximates data only after the switch, pre-switch values are identical to network model simulations. Overall, $R^{2}$ varies little because most samples switch on day 4 (see Figure 7). Samples with low incidence have poor approximations: their epidemic peaks do not reach 1% of the total population (Figure 3c), meaning the population is not homogeneous enough to support the well-mixed assumption. It can be argued that such low-incidence simulations need no acceleration, since their execution times are already short due to few infections.

**Figure 8:**
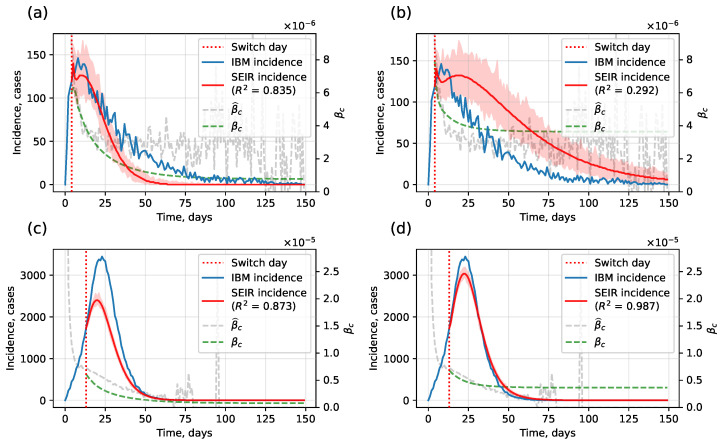
Example of regression-based and LSTM-based $\beta_{c}$ estimation: (**a**) regression, low incidence; (**b**) LSTM, low incidence; (**c**) regression, high incidence; (**d**) LSTM, high incidence.

Regression-based $\beta_{c}$ estimation, which slightly underestimates values, works better for low-incidence cases where rapid decline dominates (Figure 8). By contrast, LSTM-based estimation performs better on higher-incidence samples. Interval estimates are obtained by running multiple stochastic SEIR simulations with the same $\beta_{c}$.

As shown in Figure 3c, samples with sufficiently high peaks occupy the upper-right corner in parameter space. For these samples, the best-performing method is LSTM-based estimation (mean $R^{2}=0.5$), which is chosen for further calibration experiments.

#### 4.1.2. Surrogate Approach

The surrogate autoencoder model was trained to simulate incidence values for epidemic parameters value ranges from Table 2. The inputs were the parameter pair $\left(\alpha ,\beta_{n}\right)$, and the outputs were incidence trajectories. The optimal architecture used hidden size H=256 and latent dimension L=32, determined via grid search.

**Figure 9:**
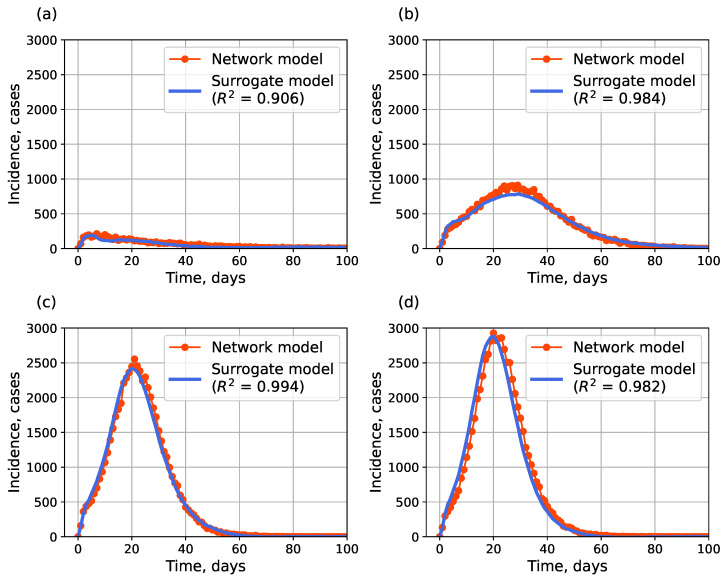
Examples of surrogate autoencoder (AE) simulations: (**a**) $\alpha =0.44,\beta_{n}=0.35$; (**b**) $\alpha =0.59,\beta_{n}=0.40$; (**c**) $\alpha =0.71,\beta_{n}=0.41$; (**d**) α=0.75,β=0.37. Barabási–Albert topology.

**Figure 10:**
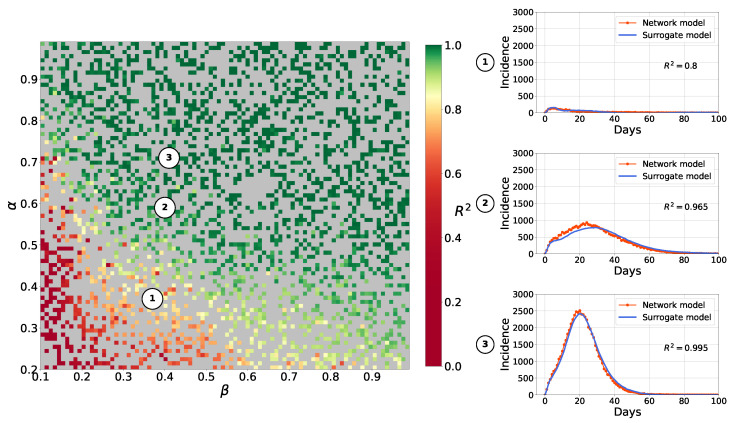
Goodness of fit of autoencoder surrogate across parameter space, Barabási–Albert network topology. Colors represent $R^{2}$ values between network- and surrogate-simulated incidence curves. Gray cells indicate training points. Circles with numbers indicate parameter values for curves. For small-world topology, see Appendix A, Figure A5.

Figure 9 compares point surrogate simulations to the network model incidence. The surrogate demonstrates high fidelity (mean $R^{2}>0.89$). The quality of fit across the parameter grid for the Barabasi–Albert topology is shown in Figure 10. The results for the small-world topology are shown in Appendix A, Figure A5.

**Figure 11:**
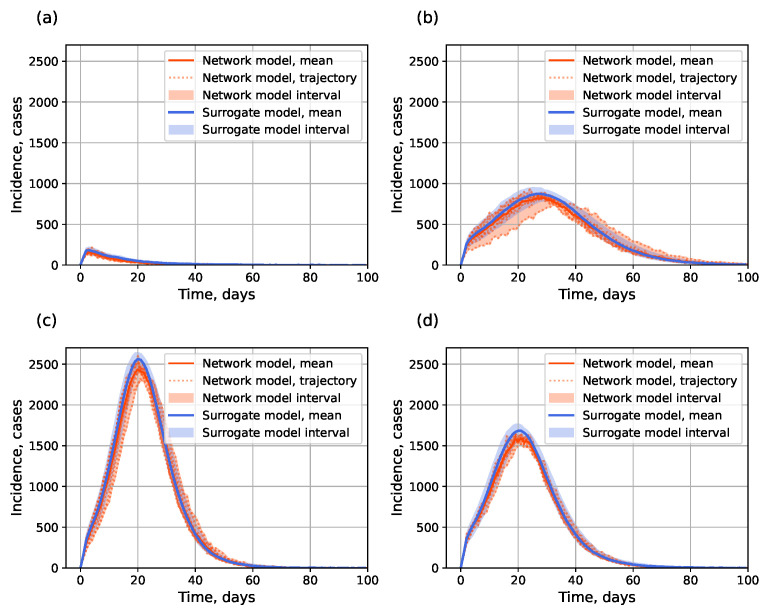
Examples simulated incidence with interval estimation using the surrogate approach: (**a**) $\alpha =0.29,\;\beta_{n}=0.84$; (**b**) $\alpha =0.59,\;\beta_{n}=0.40$; (**c**) $\alpha =0.71,\;\beta_{n}=0.41$; (**d**) $\alpha =0.6,\;\beta_{n}=0.66$. Barabási–Albert topology.

**Figure 12:**
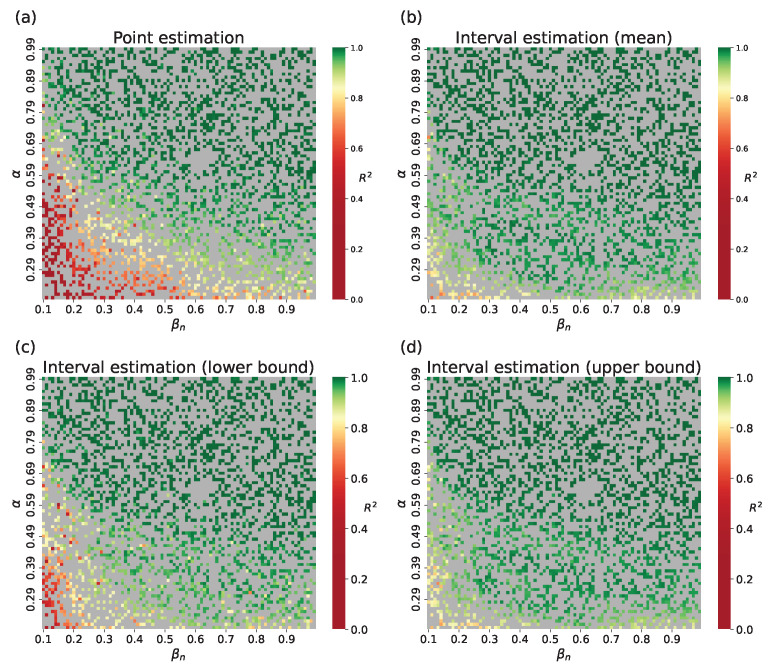
Accuracy of the surrogate approach, Barabási–Albert topology for the point assessment (replication of a single trajectory) and interval assessment (replicating the interval measured on 10 stochastic simulations of the original network model): (**a**) point estimation; (**b**) interval estimation, $R^{2}$ for mean values; (**c**) interval estimation, $R^{2}$ for lower bound values; (**d**) interval estimation, $R^{2}$ for upper bound values. Gray cells indicate training points.

**Figure A2:**
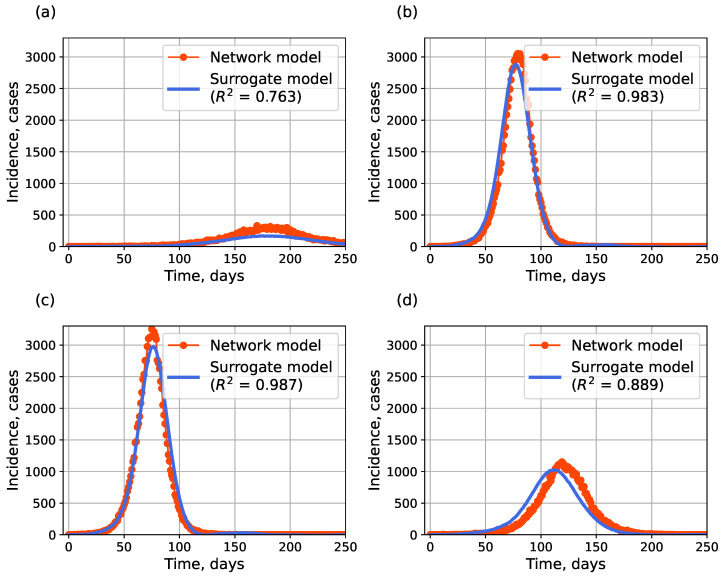
Examples of surrogate autoencoder (AE) simulations, small-world topology: (**a**) $\alpha =0.29,\beta_{n}=0.84$; (**b**) $\alpha =0.97,\beta_{n}=0.73$; (**c**) $\alpha =0.98,\beta_{n}=0.74$; (**d**) α=0.66,β=0.6.

**Figure A3:**
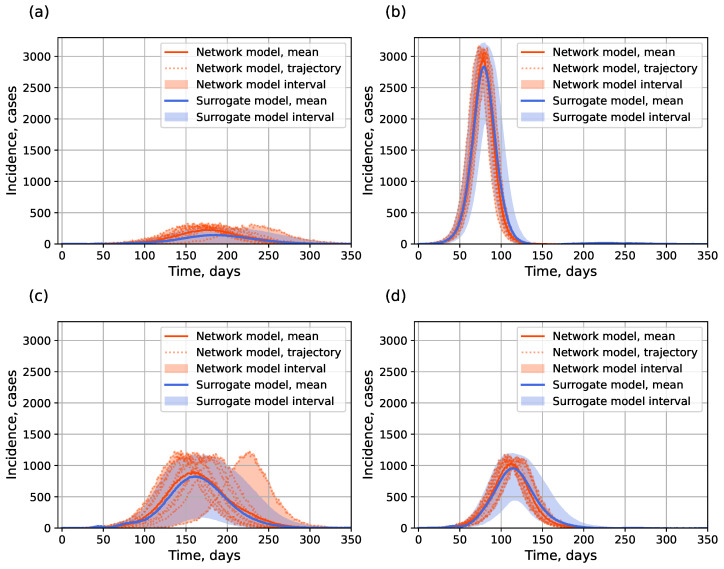
Examples simulated incidence with interval estimation using the surrogate approach, small world topology: (**a**) $\alpha =0.29,\beta_{n}=0.84$; (**b**) $\alpha =0.97,\beta_{n}=0.73$; (**c**) $\alpha =0.99,\beta_{n}=0.32$; (**d**) α=0.66,β=0.6.

**Figure A4:**
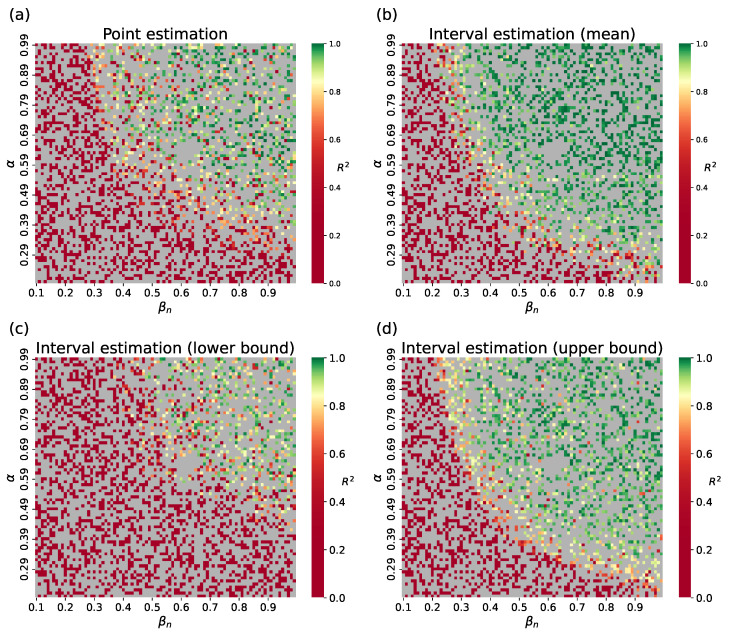
Accuracy of the surrogate approach, small-world topology, for the point assessment (replication of a single trajectory) and interval assessment (replicating the interval measured on 10 stochastic simulations of the original network model): (**a**) point estimation; (**b**) interval estimation, $R^{2}$ for mean values; (**c**) interval estimation, $R^{2}$ for lower bound values; (**d**) interval estimation, $R^{2}$ for upper bound values. Gray cells indicate training points.

**Figure A5:**
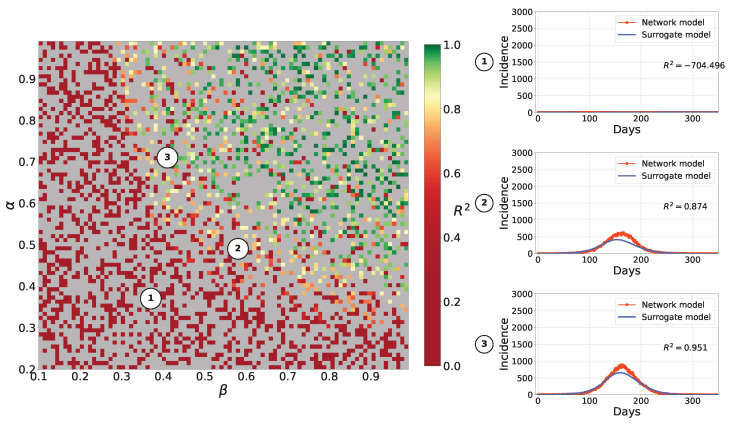
Goodness of fit of autoencoder surrogate across parameter space, small-world network topology. Colors represent $R^{2}$ values between network- and surrogate-simulated incidence curves. Gray cells indicate training points. Circles with numbers indicate parameter values for curves.

Figure 11 shows example outputs, and Figure 12 shows $R^{2}$ over the parameter space. Since mean $R^{2}$ is bigger than 0.94 for each curves of μ(t),l(t), and u(t), we can assume that the surrogate is highly accurate at capturing both mean epidemic dynamics and stochastic bounds. The results for the small-world topology are shown in Figure A2, Figure A3 and Figure A4.

### 4.2. Parameter Estimation on a Synthetic Incidence Data

#### 4.2.1. Retrospective Calibration

**Figure A6:**
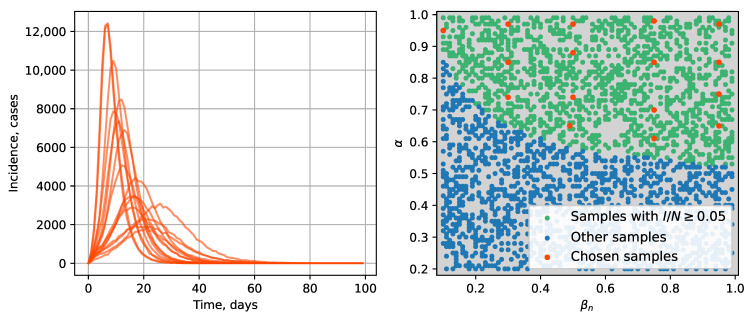
Incidence trajectories resulted from chosen parameter pairs (**left**); heatmap for chosen parameter pairs (**right**).

**Figure A7:**
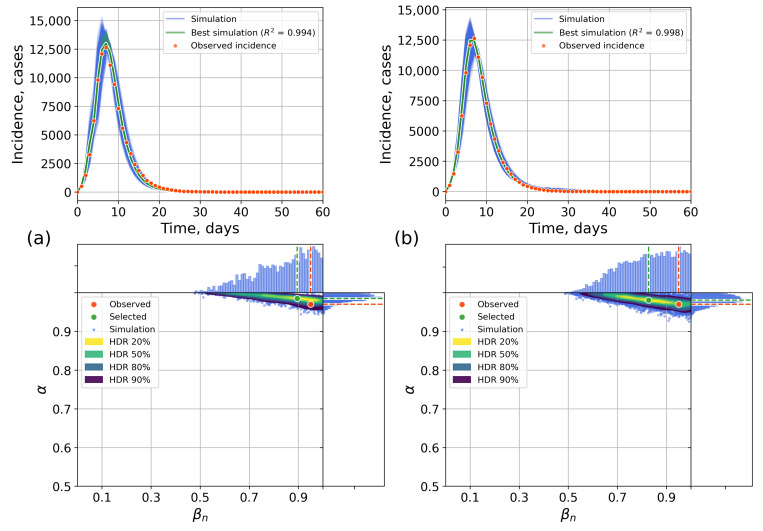
Calibration on a simulated outbreak with (**a**) the hybrid approach, (**b**) the surrogate approach. **Top**: posterior-sampled trajectories; **bottom**: posterior parameter distributions.

**Figure A8:**
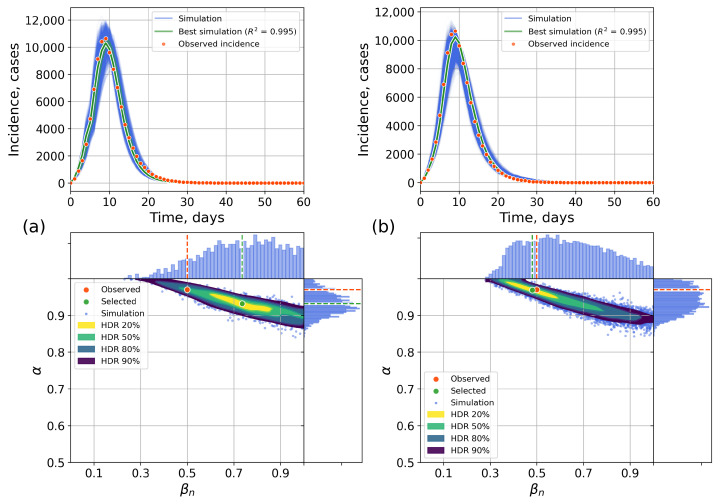
Calibration on a simulated outbreak with (**a**) the hybrid approach, (**b**) the surrogate approach. **Top**: posterior-sampled trajectories; **bottom**: posterior parameter distributions.

**Figure A9:**
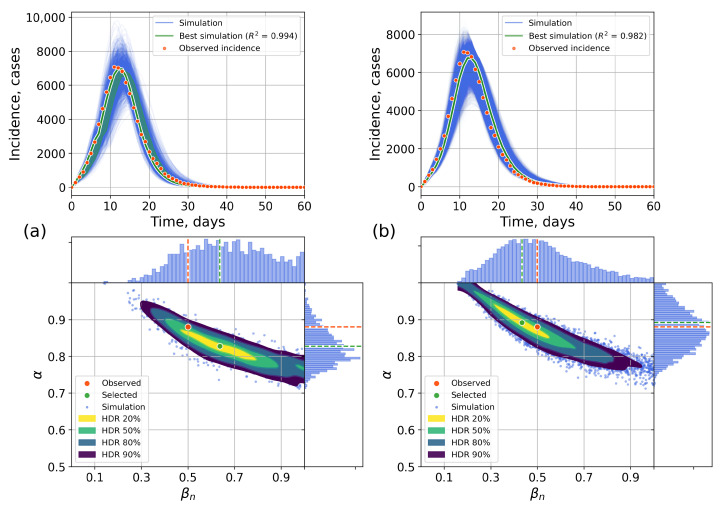
Calibration on a simulated outbreak with (**a**) the hybrid approach, (**b**) the surrogate approach. **Top**: posterior-sampled trajectories; **bottom**: posterior parameter distributions.

**Figure A10:**
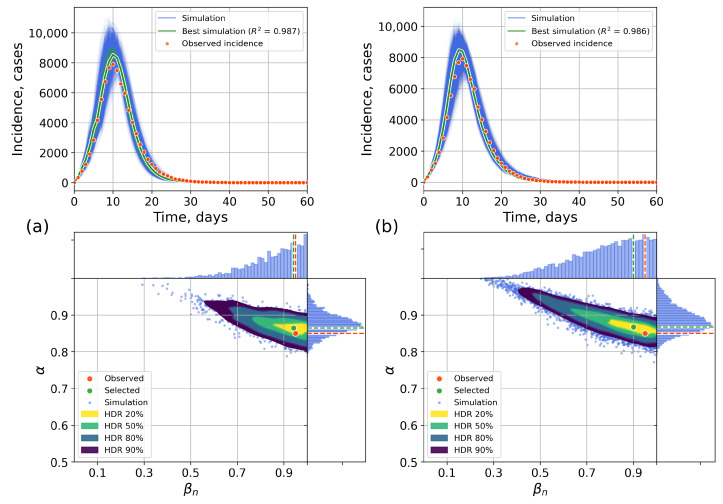
Calibration on a simulated outbreak with (**a**) the hybrid approach, (**b**) the surrogate approach. **Top**: posterior-sampled trajectories; **bottom**: posterior parameter distributions.

**Figure A11:**
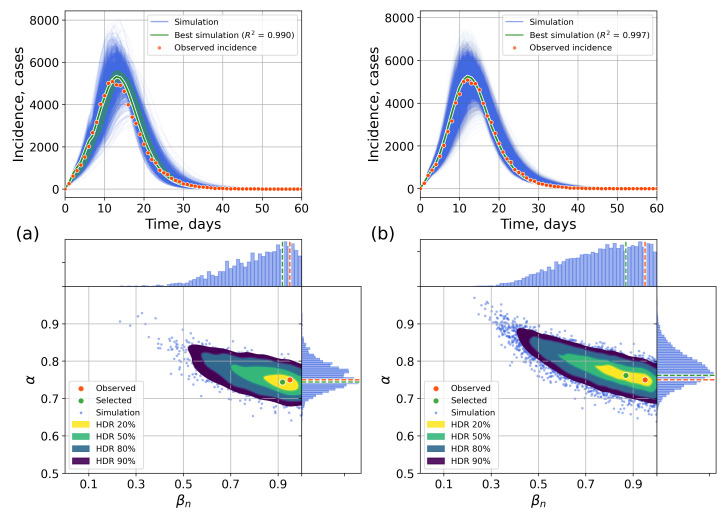
Calibration on a simulated outbreak with (**a**) the hybrid approach, (**b**) the surrogate approach. **Top**: posterior-sampled trajectories; **bottom**: posterior parameter distributions.

**Figure A12:**
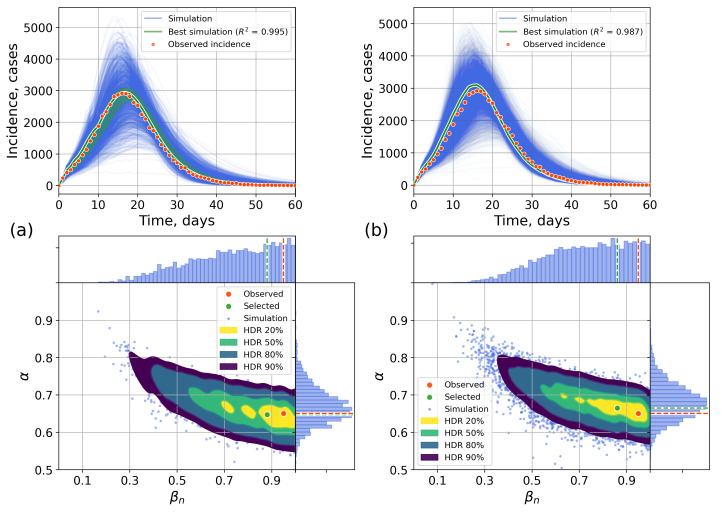
Calibration on a simulated outbreak with (**a**) the hybrid approach, (**b**) the surrogate approach. **Top**: posterior-sampled trajectories; **bottom**: posterior parameter distributions.

**Figure A13:**
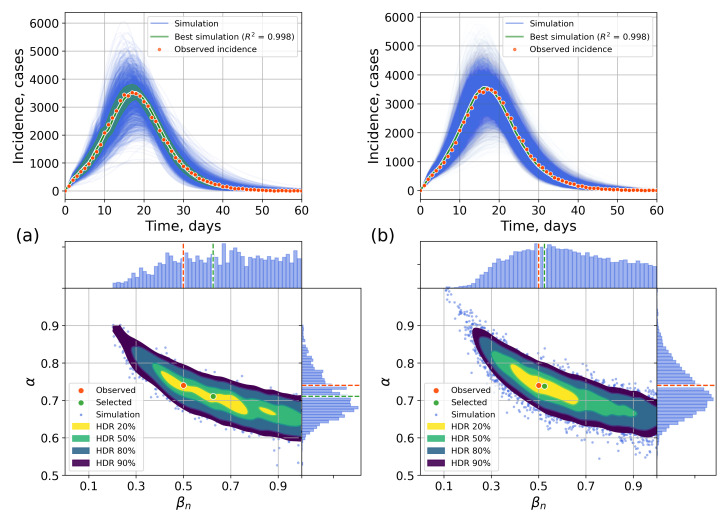
Calibration on a simulated outbreak with (**a**) the hybrid approach, (**b**) the surrogate approach. **Top**: posterior-sampled trajectories; **bottom**: posterior parameter distributions.

**Figure A14:**
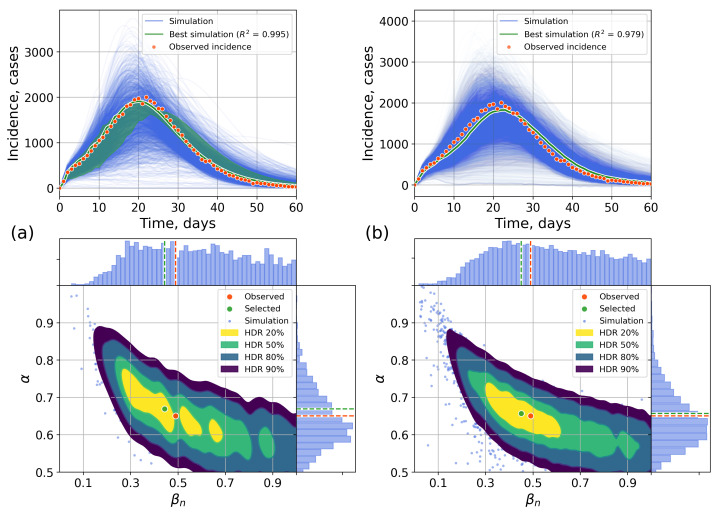
Calibration on a simulated outbreak with (**a**) the hybrid approach, (**b**) the surrogate approach. **Top**: posterior-sampled trajectories; **bottom**: posterior parameter distributions.

**Figure A15:**
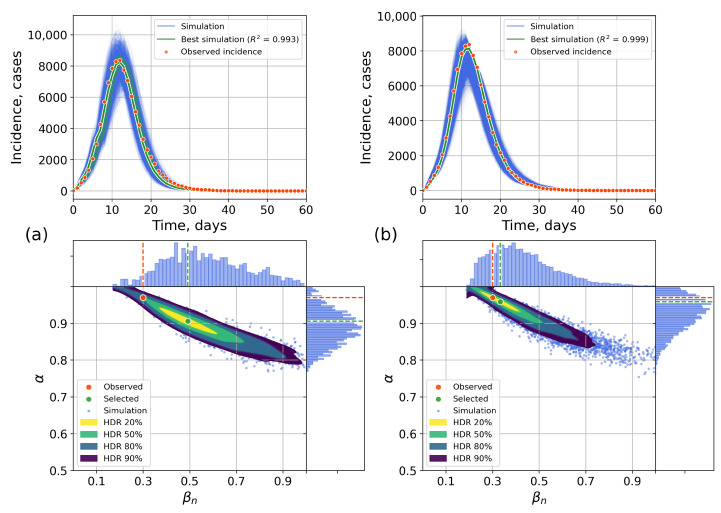
Calibration on a simulated outbreak with (**a**) the hybrid approach, (**b**) the surrogate approach. **Top**: posterior-sampled trajectories; **bottom**: posterior parameter distributions.

**Figure A16:**
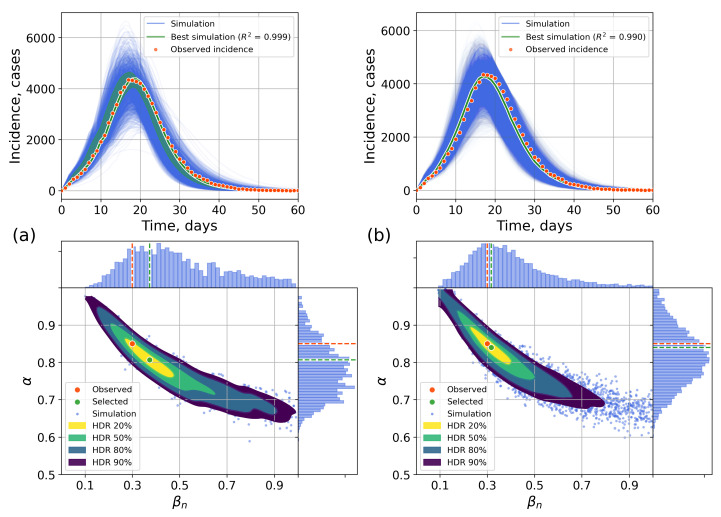
Calibration on a simulated outbreak with (**a**) the hybrid approach, (**b**) the surrogate approach. **Top**: posterior-sampled trajectories; **bottom**: posterior parameter distributions.

**Figure A17:**
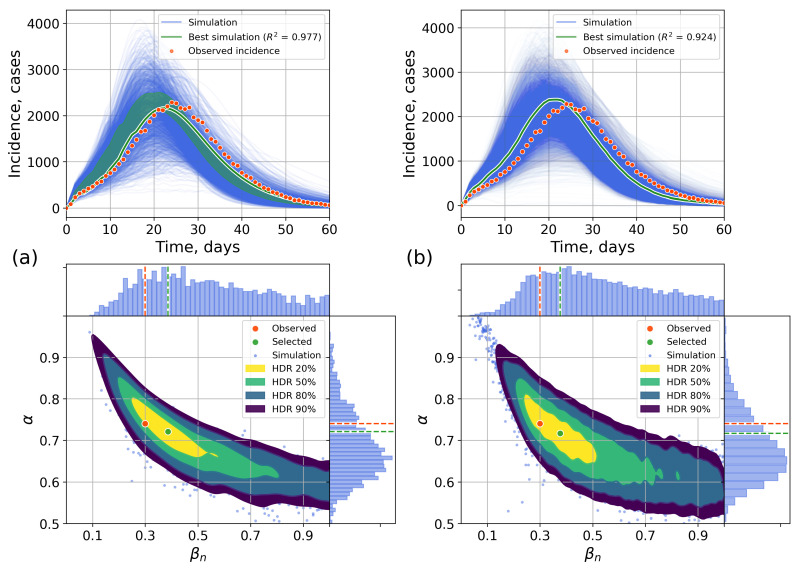
Calibration on a simulated outbreak with (**a**) the hybrid approach, (**b**) the surrogate approach. **Top**: posterior-sampled trajectories; **bottom**: posterior parameter distributions.

**Figure A18:**
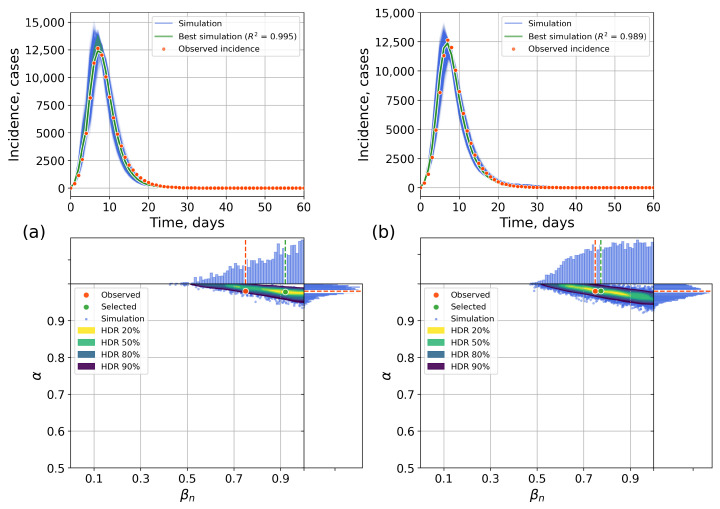
Calibration on a simulated outbreak with (**a**) the hybrid approach, (**b**) the surrogate approach. **Top**: posterior-sampled trajectories; **bottom**: posterior parameter distributions.

**Figure A19:**
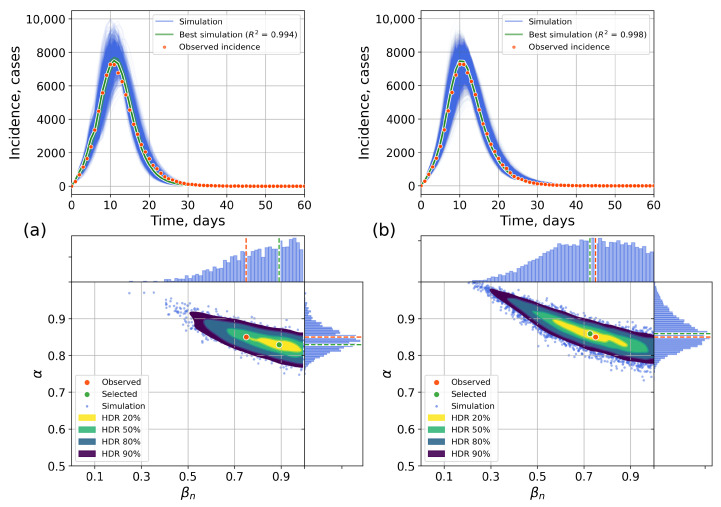
Calibration on a simulated outbreak with (**a**) the hybrid approach, (**b**) the surrogate approach. **Top**: posterior-sampled trajectories; **bottom**: posterior parameter distributions.

**Figure A20:**
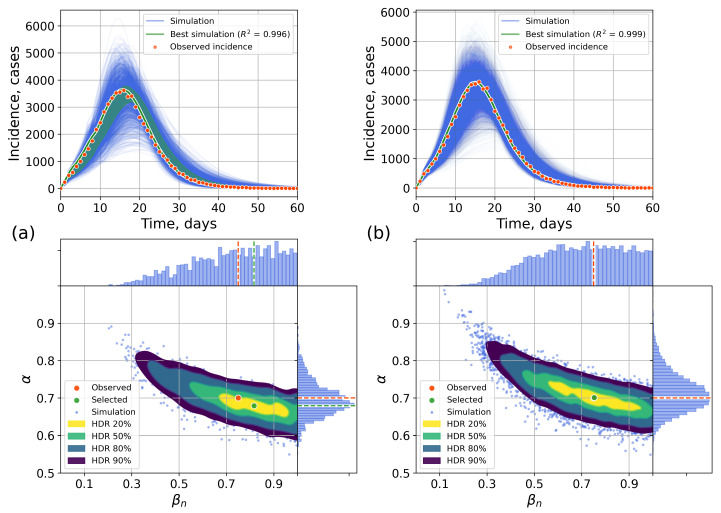
Calibration on a simulated outbreak with (**a**) the hybrid approach, (**b**) the surrogate approach. **Top**: posterior-sampled trajectories; **bottom**: posterior parameter distributions.

**Figure A21:**
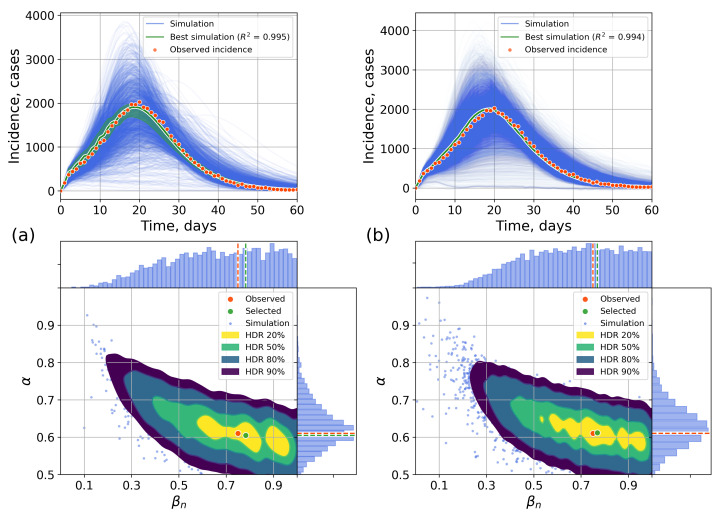
Calibration on a simulated outbreak with (**a**) the hybrid approach, (**b**) the surrogate approach. **Top**: posterior-sampled trajectories; **bottom**: posterior parameter distributions.

Next we assessed the two approaches in the task of calibrating the model parameters to a target incidence curve simulated by the network model. The target parameters were $\alpha =0.95,\beta_{n}=0.1$; the other values followed Table 2. The results for other pairs of ($\alpha ,\beta_{n}$) (Figure A6) are shown in Figure A7, Figure A8, Figure A9, Figure A10, Figure A11, Figure A12, Figure A13, Figure A14, Figure A15, Figure A16, Figure A17, Figure A18, Figure A19, Figure A20 and Figure A21. The calibration employed Approximate Bayesian Computation with Sequential Monte Carlo (ABC-SMC), implemented with the PyMC library [56]. The scaling parameter ε for computing distance between the observed and simulated trajectories (a smaller value allows less variation) was set to ε=2000.

**Figure 13:**
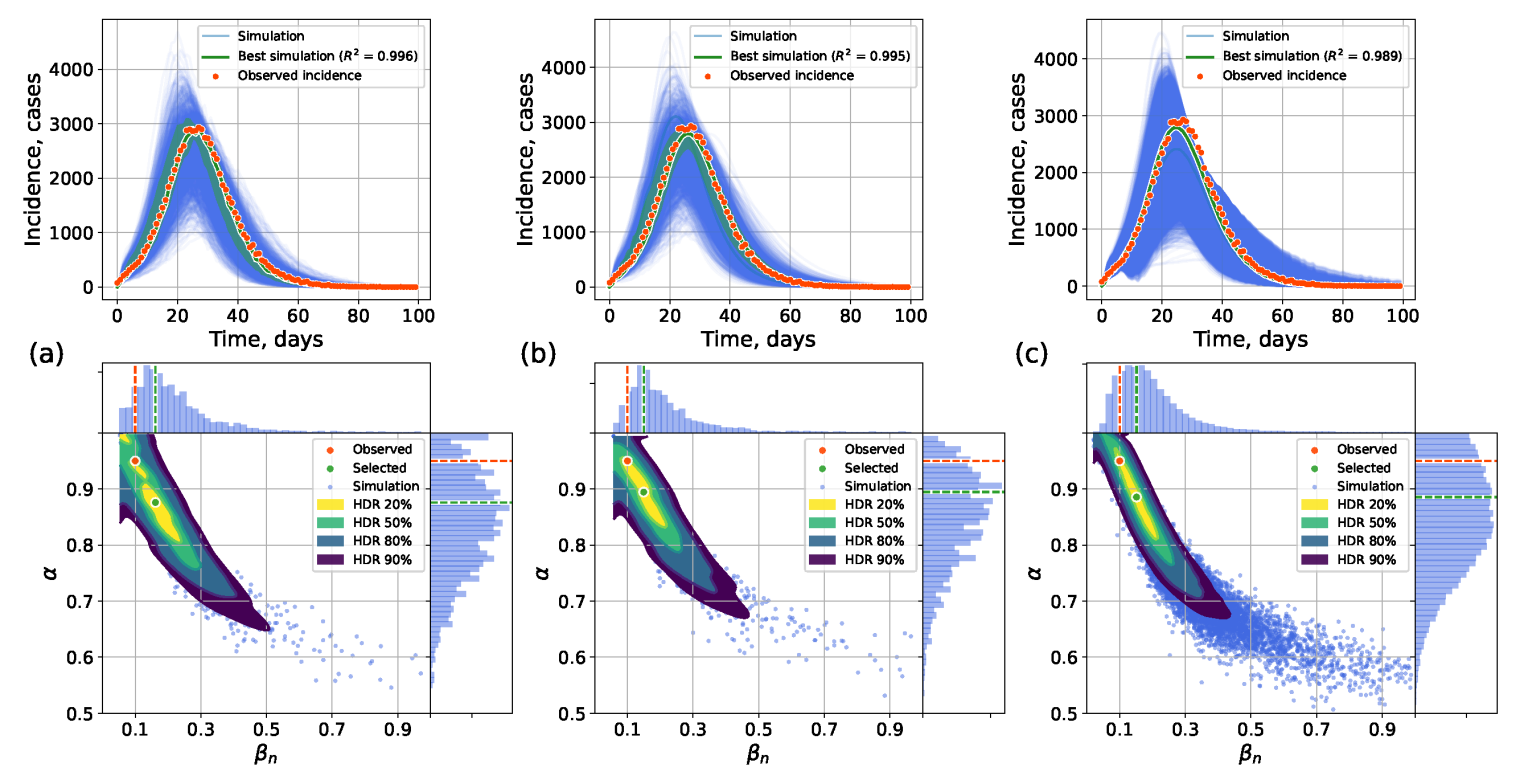
Calibration on a simulated outbreak with (**a**) the network model, (**b**) the hybrid approach, (**c**) the interval surrogate approach. **Top**: posterior-sampled trajectories; **bottom**: posterior parameter distributions.

The calibration results for the original network model serve as a baseline (Figure 13a). Execution was computationally intensive, so the hybrid and network calibrations used 600 samples across three ABC-SMC runs, while the surrogate calibration used 2000 samples across four runs. The timings are given in Table 5.

The results for the hybrid approach are shown in Figure 13b. The selected method of $\beta_{c}$ estimation was LSTM. The switch was performed when I/N≥0.05 to ensure appropriate homogeneity. The best parameters were selected as the centroid of the 20% highest density region (HDR). Interval estimates were obtained by running multiple stochastic SEIR simulations.

The calibration results for the interval surrogate approach is shown in Figure 13c.

In all the approaches (Figure 13), the posterior value assessments do not coincide with the true parameters. This reflects the parameter estimation ambiguity: different (α,β) pairs can produce similar incidence curves. For example, a high immunity level (1−α) with a high transmission rate (β) can result in the same epidemic as a low immunity level and a low transmission rate [57].

#### 4.2.2. Forecasting Examples

In this section, we demonstrate the examples of forecast generation by both proposed approaches. Typically there are two ways to apply forecasting frameworks for the aims of epidemic surveillance. The first and the most typical one is to perform short-time forecasting of the disease incidence with the time span from one to two weeks. The second one is to predict the day and height of peak incidence, which is motivated by the need of healthcare organs to assess the maximum burden imposed on healthcare units and the time left to prepare necessary resources. Here we demonstrate the results for both use cases.

During the forecasting procedure, the models are used in the same way as when calibrated to past outbreak data. The key distinction is that retrospective calibration utilizes the whole synthetic incidence dataset, whereas forecasting is performed with only the initial part of it (incidence at times $t_{0}$, $t_{1}$, …, $t_{k}<T$), which we define as “known data”. By calibrating on incomplete data, we obtain parameter value distributions and generate multiple incidence curves, from which a forecast incidence interval is constructed.

The incidence curve from Section 4.2.1 was used ($\alpha =0.95,\beta_{n}=0.1$) for the experiments. We explored three forecasting scenarios with different lengths of known incidence data: (1) $t_{k}=t_{peak}-14$; (2) $t_{k}=t_{peak}-7$; (3) $t_{k}=t_{peak}+7$. The forecast length was 14 days.

Early forecasting (14 days before the peak) can be limited in precision, as the outbreak is just beginning and low number of infected leads to highly variable outcomes. On the contrary, forecasting after the peak leads to much better accuracy of the output incidence but is less useful for the purposes of epidemic surveillance, since the moment of maximum disease intensity has already passed. The aim of our experiments was to show that in case of forecasting before the peak, our approaches are able to correctly assume the general direction of disease dynamics capturing the real incidence points by the calculated intervals of uncertainty, and in case of forecasting after the peak, they can generate forecasts with good accuracy narrowing the uncertainty.

**Figure 14:**
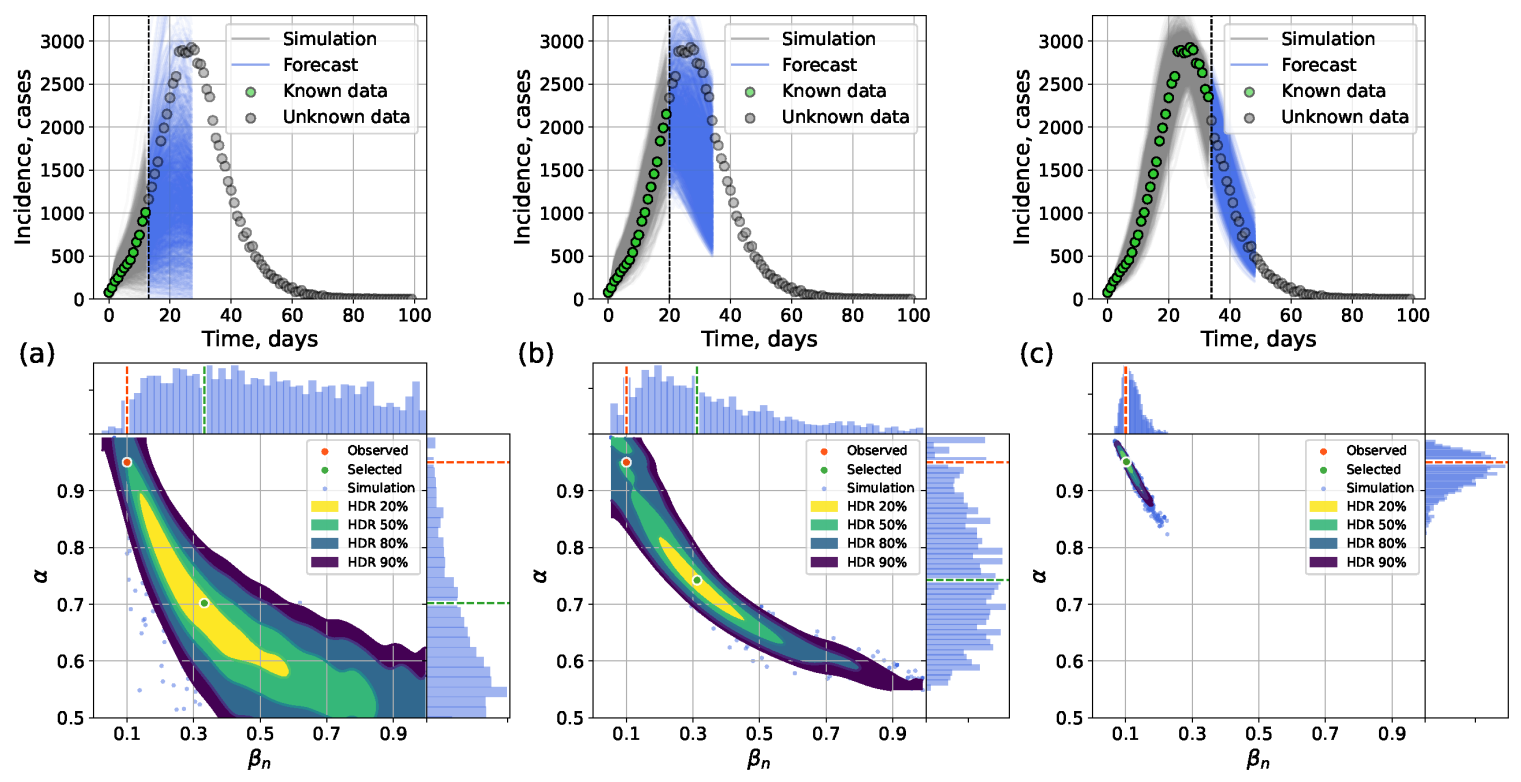
Forecasting with the hybrid approach using data available until (**a**) 14 days before the peak, (**b**) 7 days before the peak, (**c**) 7 days after the peak. **Top**: posterior-sampled trajectories; **bottom**: posterior parameter distributions.

**Figure 15:**
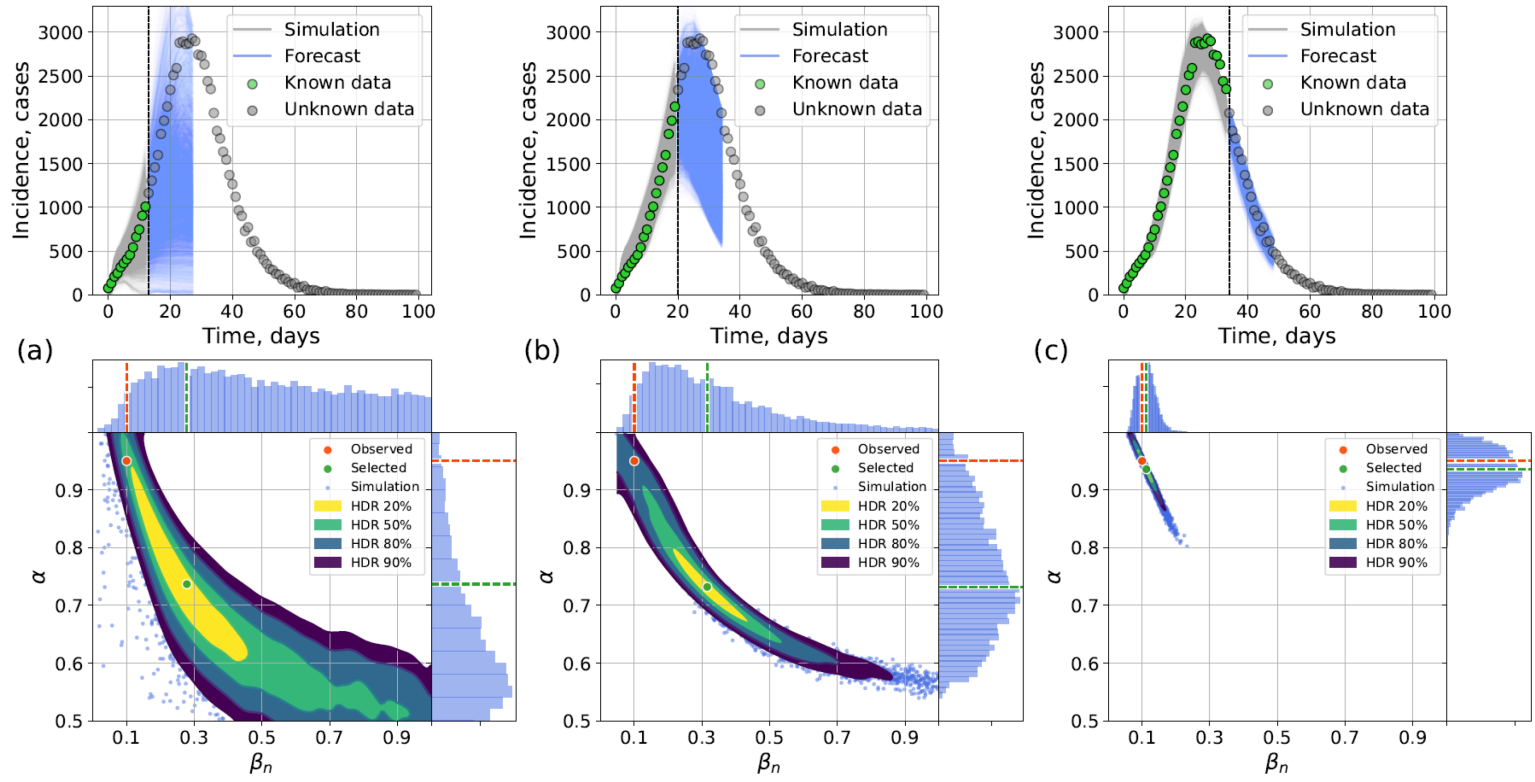
Forecasting with surrogate approach using data available until (**a**) 14 days before the peak, (**b**) 7 days before the peak, (**c**) 7 days after the peak. **Top**: posterior-sampled trajectories; **bottom**: posterior parameter distributions.

Figure 14 and Figure 15 show hybrid and surrogate forecasts with scaling parameter ε=500. The average timings for the network model, the hybrid, and the surrogate approaches are presented in Table 5. Early-stage forecasts (14 days pre-peak) admit wide uncertainty; forecasts closer to and beyond the peak are narrower and more accurate.

**Figure 16:**
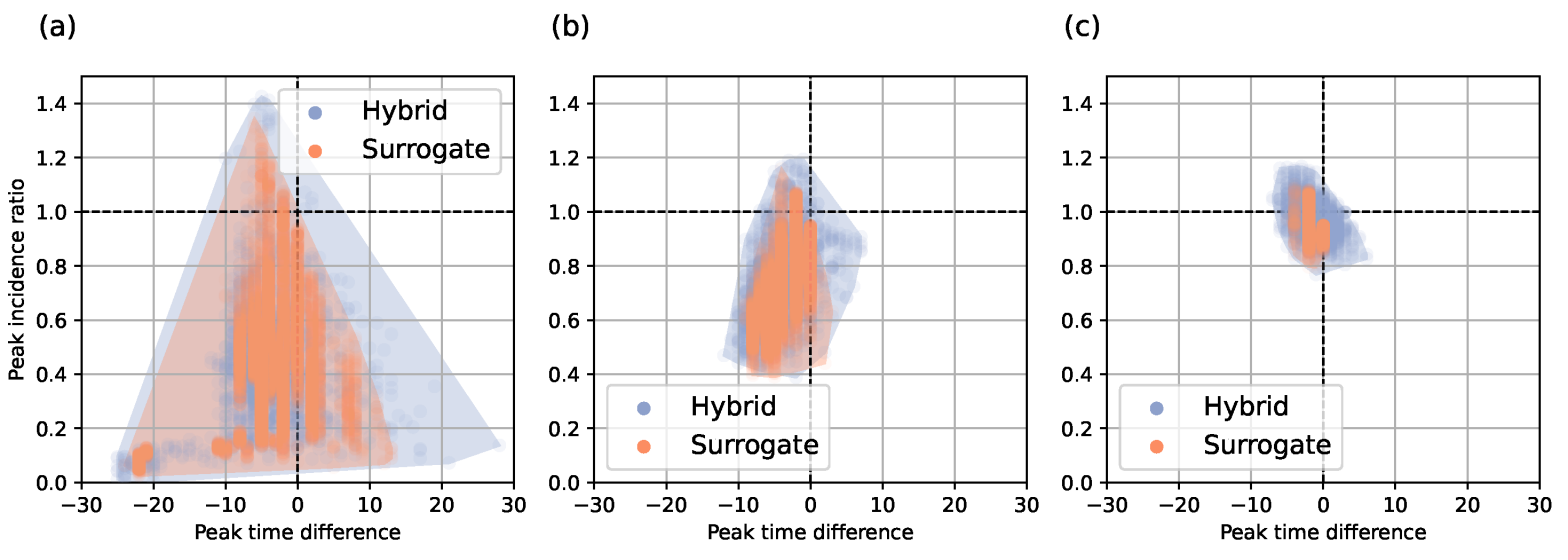
Peak errors for hybrid and surrogate approaches for forecasting using incidence data: (**a**) 14 days before peak, (**b**) 7 days before peak, (**c**) 7 days after peak.

Figure 16 shows peak errors for three cases of forecasting. The *x*-axis shows the difference between the predicted and actual peak time. The y-axis shows the fraction of the predicted peak incidence to the actual peak incidence. The best case is at the point with coordinates (0, 1). The second and third quadrants depict cases with peaks predicted earlier, which is preferable: if peak time error is positive, we will be caught by surprise unprepared.

## 5. Discussion

In this work, we evaluated two complementary approaches for accelerating epidemic forecasting with detailed epidemic models: the hybrid approach that couples a network model with a compartmental model, and the surrogate approach that emulates epidemic dynamics using an autoencoder model trained on network data. Both approaches were tested on synthetic data to measure their accuracy in tasks of calibration to real data and forecasting. The hybrid approach benefits from ML-based $\beta_{c}$ estimation methods such as LSTM, yielding more accurate parameter projections than purely statistical estimators. The surrogate approach bypasses explicit simulation altogether by training ML models to reproduce epidemic outputs, thus offering efficiency gains. The presented methods facilitate forecasting with detailed epidemic models and, being used within a single modeling framework, help compensate for the drawbacks of each other.

### 5.1. Methods Advantages and Drawbacks

The hybrid approach enables a twofold acceleration of average simulation time and a 1.6-fold acceleration in calibration (Table 5) compared with the network model. Replacing statistical estimators (e.g., last $\beta_{c}$ value) with ML estimators further reduces RMSE by a factor of three (Table 3), with LSTM consistently outperforming alternatives.

The hybrid framework has three important advantages:

- Independence from training data at the outbreak onset with certain $\beta_{c}$ estimators. Two statistical $\beta_{c}$ estimators, last $\beta_{c}$ and cumulative $\beta_{c}$, require only the knowledge of the contact network topology and δ and γ values related to the disease properties, all of which are known/can be calculated during epidemic onset. Thus these statistical estimators can be applied immediately, making this approach suitable for early epidemic stages with limited prior information.
- Preservation of explicit stochasticity. Unlike the surrogate approach which produces a single deterministic output (point or interval assessment) for a fixed input parameter set, the hybrid approach is capable of generating multiple trajectories for a fixed input to reflect the influence of random factors, just like the original IBM it aims at replacing—by means of the actual IBM before the switch or via the stochastic SEIR model after the switch.
- Interpretability. It is possible to use different $\beta_{c}$ trajectories to analyze the impact of government policies such as lockdowns, or to assess future $\beta_{c}$ values.

The main disadvantages are as follows:

- Homogeneity assumption. When incidence remains very low, the SEIR submodel is ill-suited, and switching produces poor approximations.
- Computational cost relative to surrogate modeling. Although faster than the pure network model, the hybrid approach is still slower than the surrogate approach.

The surrogate approach accelerates simulations by up to $6.8\times 10^{4}$ times (Table 5), without substantial loss in accuracy. This is especially valuable in calibration and forecasting contexts, where thousands of repeated runs are required. Furthermore, the surrogate approach can be trained to capture stochastic variability by predicting lower and upper bounds of epidemic trajectories. Additional compartments can also be modeled, making the surrogate flexible and extensible.

Training the autoencoder-based surrogate required less than 10 min. However, generating its training dataset with the individual-based model required approximately 80 CPU hours. Thus, while the surrogate is efficient once trained, initial data generation remains a major bottleneck.

### 5.2. Application to Real Epidemic Data

The application of the presented modeling approaches in real epidemic surveillance assumes the following scenario:

- There exists an incomplete incidence dataset $\left\{I_{0}^{new},I_{1}^{new},I_{2}^{new},\ldots I_{k}^{new}\right\}$.
- After each time period (day or week) passed, a new measurement $I_{k+1}^{new}$ is added to the dataset.

The aims of the model application are as follows:

- Perform short-term prediction of disease incidence and peak height/time prediction
- Simulate the effect of control measures on the prediction

A pragmatic workflow could proceed as follows:

- Begin with the network model for an entirely new outbreak.
- Once infection prevalence rises sufficiently, employ the hybrid approach with statistical $\beta_{c}$ estimation to provide early forecasts.
- After accumulating sufficient simulation data and/or obtaining incidence data from similar cities where the epidemic has already passed, train the surrogate model, which can then take over for large-scale parameter calibration and forecasting.

This strategy leverages the immediate applicability of the hybrid approach with the long-term efficiency of the surrogate approach.

The control measures can be mimicked by the models in the following fashion:

- Quarantine measures can be simulated by assuming slower transmission due to fewer contacts. On the level of models, it is made by changing $\beta_{c}\left(t\right)$ in the SEIR part of the hybrid approach and change of $\beta_{n}$ in the surrogate approach. In its current implementation, the surrogate does not support a variable $\beta_{n}$, so it can be used only for playing “what-if” scenarios when change in $\beta_{n}$ happened before the outbreak (for instance, quarantine measures were introduced before the inception of the virus in the population). This might be changed in its future implementations to allow dynamical adjustment of trajectories during the simulation.
- Real-time vaccination may be considered within a hybrid approach by changing $\beta_{c}\left(t\right)$. In a surrogate approach, vaccination is simulated by changing the proportion of immune individuals in the population, i.e., generally, the vaccination campaign is assumed to be finished before the outbreak.

The given scenarios are approximate, as the models were not explicitly tested in the described settings. We plan to correct the application scenarios as a result of model calibration to real data.

### 5.3. Study Limitations

The study has the following limitations:

- According to the assumptions stated, this study considers a population whose contact patterns can be reasonably approximated by a static, regular network topology. Disease properties are also assumed to be constant. As a result, this study does not cover other important cases, such as several moderately interconnected cities with Barabási–Albert or small-world networks, multiwave epidemics, or co-circulation of multiple virus strains (e.g., different SARS-CoV-2 variants or influenza strains H1N1(pdm09) and H3N2).
- The contact network structure in the city is assumed to be stable over a multi-year period, otherwise establishing a specific topology (Barabasi–Albert or small world) would be infeasible. While this is a simplification compared to real dynamic contact patterns, it is a common assumption in data-driven demographic and epidemic modeling (see, for instance, [58]), because to construct realistic dynamical contact networks, extensive and often unavailable data is required.
- We do not consider asymptomatic cases and under-reporting. While it does not affect the forecasting accuracy, which is our main interest in this study, it will lead to wrong assessment of epidemic indicators, such as effective reproduction number, when the framework is calibrated to real data. This issue will be fixed in the future studies.
- The choice of switch condition in the hybrid approach remains heuristic. Different $\beta_{c}$ estimation methods may favor different thresholds: for example, the last $\beta_{c}$ method benefits from higher prevalence thresholds, whereas LSTM performs well at earlier switches.
- Calibration with ABC-SMC requires the choice of ε. A smaller ε may narrow parameter range, but it requires more simulation time, which may limit our exploration under computational constraints.
- We tested our approaches on synthetic data, whereas real surveillance data may contain noise, delays, under-reporting, and gaps. We plan to address this issue in the future studies by using data imputation and bootstrapping techniques [59].

### 5.4. Additional Capabilities

There exist several capabilities that could increase the usefulness of the both hybrid and surrogate approaches. For one, the network models can take into account the demographic characteristics of different individuals attributed to network nodes from the records of synthetic populations [49]. Age-dependent transmission rates by age groups can also be integrated into models, for instance, in a form of contact intensity matrices [48,58]. The surrogate model can be trained on datasets created via a network model based on synthetic populations. The surrogate approach can be trained not only on incidence, but also on $\beta_{c}$ trajectories to forecast both future values of $\beta_{c}$ and the evolution of compartmental states.

### 5.5. Future Directions

Future efforts will be directed toward incorporating the presented approaches into epidemic surveillance for acute respiratory infections. This entails side-by-side comparisons with applied compartmental models using real-world data. The dataset available to us from public health includes records from a district of Saint Petersburg during the COVID-19 outbreak, containing address, sex, and age information. As noted in [60], COVID-19 transmission in urban districts is influenced, among other things, by the number of food services, bus stops, drug stores in the neighborhood. Therefore, our data would enable the fine-grained display and modeling of disease transmission given specific urban amenities, and their impact based on age, sex, and living proximity. We plan to verify the resulting models using aggregate data on COVID-19 and ARI incidence available from Research Institute of Influenza.

Since the surrogate approach demonstrated the highest efficiency, we will explore alternative neural architectures and dimensionality-reduction strategies. For example, epidemic trajectories may be generated by embedding a small number of latent parameters into the autoencoder space, offering both interpretability and efficiency for practical use in real-time epidemic response.

The application of multiscale individual-based modeling approaches to real infection data suggests the choice of a contact topology representing a real city. Based on available historic data of influenza incidence, we plan to analyze whether the shape of epidemic dynamics for one city throughout the years indicates the intrinsic contact topology for this city. If we were able to connect topology types with typical incidence curve forms, the clusterisation of Russian cities based on disease transmission patterns and hence interchangeable use of historical data within one cluster becomes a possibility.

Our detailed models and contact topology analysis can be useful for implementing another method suitable for purposes of epidemic forecasting and disease control—that is, data-driven source detection [61], which is used to identify the patient zero, a rumor spreader, or a source of pollution. Cases relevant to epidemic modeling in urban environments include multiple source detection [62] and influential/superspreader detection [63], operating under time-varying topology [64]. These technologies can enhance Russian infection surveillance and make it possible to apply approaches similar to those implemented, for instance, in SMART Pilgrim city of Makkah program, where a set of digital tools and communication technologies were deployed to forecast incidence in different “what-if” scenarios, manage crowds, and enforce social distancing to minimize the spread of the COVID-19 virus [65]. The program required all pilgrims to keep using the Tetamman application and wear their electronic tracking bands during and even after Hajj rituals were completed. Pilgrims were not just monitored through the app; they also received regular phone calls asking about their symptoms. Using these data to monitor incoming tourist flows and coupling the results of analysis with predictive models built on top of networks, seems to us a good example of how new digital technologies can help obtain fast and accurate disease forecasting and thus improve safety of citizens and population health.

## Figures and Tables

**Table 1:** SEIR model parameters and their descriptions.

Parameter	Description
α	Initial fraction of non-immune individuals
$\beta_{c},\beta_{n}$	Infection transmission rate (compartmental and network models)
1/γ	Mean latent (exposed) period, days
1/δ	Mean infectious period, days
$S_{t}$	Number of susceptible individuals on day *t*
$E_{t}$	Number of exposed individuals on day *t*
$I_{t}$	Number of infectious individuals on day *t*
$R_{t}$	Number of recovered individuals on day *t*
*N*	Total population size
*T*	Simulation time

**Table 2:** SEIR model parameter types and values.

Parameter	Type	Value
α	Varied	[0.2, 1]
$\beta_{n}$	Varied	[0.1, 1]
1/γ	Fixed	1/0.3≈3.3 days
1/δ	Fixed	1/0.2=5 days
*N*	Fixed	$10^{5}$ individuals

**Table 3:** RMSE of incidence across different switch-point thresholds and $\beta_{c}$ estimation methods; Barabási–Albert topology. Best results are colored green, worst results are colored red.

$\beta_{c}$ Estimation Method	Switch Condition: Percent of the Population				
	3%	4%	5%	6%	7%
Last Value	358.44±202.22	356.73±203.05	357.54±209.13	356.56±211.70	356.46±215.11
Cumulative Average	2765.22±3065.45	2841.24±3126.44	2926.33±3204.48	3023.50±3325.37	3102.23±3432.34
Median	316.64±353.69	295.19±329.05	274.80±305.71	253.29±276.09	234.84±250.37
Regression	343.35±368.48	296.47±317.56	263.27±282.04	233.61±242.71	212.57±213.02
LSTM	122.55±125.20	113.08±116.99	106.62±111.20	101.18±104.09	96.62±97.96
Switch day	6.66±4.80	6.84±4.89	6.93±4.87	6.96±4.86	6.94±4.86

**Table 4:** RMSE of incidence across different switch-point thresholds and $\beta_{c}$ estimation methods; small-world topology. Best results are colored green, worst results are colored red.

$\beta_{c}$ Estimation Method	Switch Condition: Percent of the Population				
	3%	4%	5%	6%	7%
Last value	93.22±108.87	102.15±120.91	114.15±136.51	129.69±154.73	145.58±172.25
Cumulative Average	92.70±81.47	99.42±88.69	107.30±98.33	118.52±112.07	129.99±126.18
Median	278.18±290.33	275.02±281.53	276.69±277.93	285.18±279.50	295.78±283.55
Regression	124.87±119.33	118.30±108.33	118.58±107.96	124.86±116.50	133.94±129.47
LSTM	172.74±200.15	194.11±213.08	216.11±223.87	240.14±233.62	260.60±238.07
Switch day	45.65±50.78	39.6±47.08	34.28±43.91	28.85±40.21	24.32±36.5

**Table 5:** Average simulation and calibration time for different approaches.

Modeling Approach	Simulation Time	Calibration Time
Network model	$6.8\times 10^{3}$ ms	$150\times 10^{3}$ s ≈ 43 h
Hybrid approach	$3.5\times 10^{3}$ ms	$91\times 10^{3}$ s ≈ 25 h
Surrogate approach	0.1 ms	50 s

## Data Availability

The code for the surrogate approach is publicly released at https://github.com/korzin-andrey/hybrid_surrogate (accessed on 14 November 2025). Other data that support the findings of this study are available upon reasonable request.

## Symbols and Abbreviations

The following symbols and abbreviations are used in this manuscript:

Symbols	
α	Fraction of non-immune individuals on day *t* = 0
$\beta_{c}$	Infection transmission rate for compartmental model
$\beta_{n}$	Infection transmission rate for network model
1/γ	Mean latent (exposed) period, days
1/δ	Mean infectious period, days
*t*	Time (days)
$S_{t}$	Number of susceptible individuals on day *t*
$E_{t}$	Number of exposed individuals on day *t*
$I_{t}$	Number of infectious individuals on day *t*
$R_{t}$	Number of recovered individuals on day *t*
$I^{\left(new\right)}\left(t\right)$	Number of new incidence cases on day *t*
*N*	Total population size
*H*	Hidden layer size for autoencoder model
*B*	Batch size during training autoencoder model
*L*	Latent vector size in autoencoder model
Abbreviations	
ABM	Agent-based model
AE	Autoencoder
ARI	Acute respiratory infection
COVID-19	Coronavirus disease 2019
HDR	Highest density region
IBM	Individual-based model
LSTM	Long short-term memory
ML	Machine learning
SEIR	Susceptible–exposed–infected–recovered
SIR	Susceptible–infected–recovered

## Appendix C. The Network Model Description

- Input:
  – A graph G=(V,E) with |V|=N nodes, representing individuals and their contact patterns.
  – Disease progression parameters: incubation rate γ, recovery rate δ, and a base transmission rate $\beta_{n}$.
  – The initial number of infected individuals, $I_{0}$.
  – The proportion of the population with prior immunity, α.
- Output:
  – Time series of compartment counts: $S_{t},E_{t},I_{t},R_{t}$ for t=0,1,…,T.
  – (Optional) a detailed event log specifying the state of each node at each time *t*.
- Simulation procedure:
  1. Initialization:
    – Assign prior immunity: Uniformly at random, assign R(0)=⌊(1−α)×N⌋ nodes to the Recovered (*R*) state.
    – Seed initial infection: From the remaining susceptible nodes, uniformly at random assign $I\left(0\right)=I_{0}$ nodes to the infected (*I*) state. All the other non-immune nodes are set to Susceptible (*S*). The Exposed (*E*) compartment is initially empty.
    – Set the initial simulation time t←0.
  2. Main loop: while t<T:
    – For each node in state *E*, schedule an E→I event time by sampling from Exp(γ).
    – For each node in state *I*, schedule an I→R event time by sampling from Exp(δ).
    – For each node in state *S* with one or more infected neighbors, calculate the cumulative force of infection $\lambda =\beta_{n}\times (\mathrm{number}\;\mathrm{of}\;\mathrm{infected}\;\mathrm{neighbors})$. Schedule an S→E event time by sampling from Exp(λ).
    – Find the next event: Determine $t_{\mathrm{next}}$, the minimum time among all scheduled events.
    – Advance time: Set $t\leftarrow t_{\mathrm{next}}$.
    – Execute event: Change the state of the node associated with the event at $t_{\mathrm{next}}$.
      * If S→E: Remove the node from *S*, add to *E*. Schedule a new E→I event for it.
      * If E→I: Remove the node from *E*, add to *I*. Schedule a new I→R event for it.
      * If I→R: Remove the node from *I*, add to *R*.
    – Update the scheduled events list: Remove the executed event from scheduled events.
  3. Termination: the simulation concludes at time *T*. The time series $S_{t},E_{t},I_{t},R_{t}$ is returned.
